# Resolvin D1 ameliorates Inflammation-Mediated Blood-Brain Barrier Disruption After Subarachnoid Hemorrhage in rats by Modulating A20 and NLRP3 Inflammasome

**DOI:** 10.3389/fphar.2020.610734

**Published:** 2021-02-03

**Authors:** Chengcong Wei, Shenquan Guo, Wenchao Liu, Fa Jin, Boyang Wei, Haiyan Fan, Hengxian Su, Jiahui Liu, Nan Zhang, Dazhao Fang, Guangxu Li, Shixing Shu, Xifeng Li, Xuying He, Xin Zhang, Chuanzhi Duan

**Affiliations:** ^1^ Neurosurgery Center, Department of Cerebrovascular Surgery, The National Key Clinical Specialty, Engineering Technology Research Center of Education Ministry of China on Diagnosis and Treatment of Cerebrovascular Disease, Guangdong Provincial Key Laboratory on Brain Function Repair and Regeneration, The Neurosurgery Institute of Guangdong Province, Zhujiang Hospital, Southern Medical University, Guangzhou, China; ^2^ Department of Neurosurgery, Minzu Hospital of Guangxi Zhuang Autonomous Region, Affiliated Minzu Hospital of Guangxi Medical University, Nanning, China

**Keywords:** resolvin D1, A20, NLRP3 inflammasome, blood-brain barrier, inflammation, subarachnoid hemorrhage

## Abstract

Inflammation is typically related to dysfunction of the blood-brain barrier (BBB) that leads to early brain injury (EBI) after subarachnoid hemorrhage (SAH). Resolvin D1 (RVD1), a lipid mediator derived from docosahexaenoic acid, possesses anti-inflammatory and neuroprotective properties. This study investigated the effects and mechanisms of RVD1 in SAH. A Sprague-Dawley rat model of SAH was established through endovascular perforation. RVD1was injected through the femoral vein at 1 and 12 h after SAH induction. To further explore the potential neuroprotective mechanism, a formyl peptide receptor two antagonist (WRW4) was intracerebroventricularly administered 1 h after SAH induction. The expression of endogenous RVD1 was decreased whereas A20 and NLRP3 levels were increased after SAH. An exogenous RVD1 administration increased RVD1 concentration in brain tissue, and improved neurological function, neuroinflammation, BBB disruption, and brain edema. RVD1 treatment upregulated the expression of A20, occludin, claudin-5, and zona occludens-1, as well as downregulated nuclear factor-κBp65, NLRP3, matrix metallopeptidase 9, and intercellular cell adhesion molecule-1 expression. Furthermore, RVD1 inhibited microglial activation and neutrophil infiltration and promoted neutrophil apoptosis. However, the neuroprotective effects of RVD1 were abolished by WRW4. In summary, our findings reveal that RVD1 provides beneficial effects against inflammation-triggered BBB dysfunction after SAH by modulating A20 and NLRP3 inflammasome.

## Introduction

Subarachnoid hemorrhage (SAH) is a severe life-threatening acute cerebrovascular disease with a high rate of mortality and disability (Macdonald and Schweizer, 2017; Zhang et al., 2018). Increasing research suggests that early brain injury (EBI) occurring within 72 h of SAH is responsible for the disease prognosis (Zhang et al., 2016; W. Xu et al., 2019a). However, the exact mechanism of EBI after SAH remains unclear, and there is a lack of effective therapeutic agents. Inflammation and blood-brain barrier (BBB) disruption are thought to contribute to EBI (Sehba et al., 2012; Suzuki, 2015). Hence, reduction of inflammation and stabilization of the integrity of the BBB using pharmacological agents may attenuate EBI after SAH.

It is well known that maintenance of BBB integrity is essential for the regulation of central nervous system (CNS) homeostasis (Sweeney et al., 2019). An excessive inflammatory response disrupts the junctional complex in the BBB, leading to BBB dysfunction in several inflammatory CNS diseases, including trauma, Alzheimer's disease, and SAH (Chen et al., 2014; Bennett et al., 2020; Kim et al., 2020). The destroyed BBB allows peripheral neutrophils and other immune cells to infiltrate the CNS (Huang et al., 2020). Additionally, studies have confirmed that activated microglia and recruited neutrophils release various pro-inflammatory factors and exacerbate the inflammatory response after SAH (Ye et al., 2018; Tao et al., 2019).

The D-series of resolvin molecular family is derived from the metabolism of essential fatty acids, which play a critical role in orchestration of the resolution of inflammation and in restoration of tissue homeostasis (Chiang and Serhan, 2017). Resolvin D1 (RVD1), being one of the most crucial molecules in this family, has been shown to play a dual role in the inflammatory response, the roles being anti-inflammatory and pro-resolution in nature (Serhan et al., 2008). Specifically, RVD1 limits leukocyte infiltration, counter-regulates proinflammatory factors, and enhances macrophage efferocytosis and clearance of cellular debris (Duffield et al., 2006). Furthermore, RVD1 upregulates the expression of tight junction proteins and decreases vascular permeability in lipopolysaccharide-induced cultured human endothelial cells through the IκBα pathway (Zhang et al., 2013). This suggests that RVD1 inhibits inflammation-induced disruption of the BBB. However, whether RVD1 can ameliorate inflammation and BBB disruption after SAH and its specific mechanisms remain unclear.

Formyl peptide receptor 2 (FPR2), an endogenous receptor of RVD1, is a member of the formyl peptide receptor family (Dufton and Perretti, 2010), which are enriched in the CNS and play an important role in neuroinflammatory processes (Ho et al., 2018; R. Fusco et al., 2020a). Notably, FPR2 agonism often contributes to reducing inflammation in neurological disorders, such as in SAH (Dufton and Perretti, 2010; Guo et al., 2016). The nuclear factor (NF)-κB signaling pathway plays an important role in regulating inflammation by FPR2 (Li et al., 2020). A20/tumor necrosis factor (TNF)-α-induced protein 3, also known as TNFAIP3, is a key molecule that inhibits NF-κB pathway activation by ubiquitination and exhibits significant anti-inflammatory effects in various diseases (Wertz et al., 2004; Ma and Malynn, 2012; Lu et al., 2019). Additionally, it has been demonstrated that D-series of resolvin molecules inhibit NF-κB activation by upregulating A20 expression to produce anti-inflammatory effects (Sham et al., 2018). However, the precise role of A20 in EBI after SAH is unclear. NLRP3 inflammasome activation mediates caspase-1 cleavage and secretion of interleukin (IL)-18 and IL-1β, thereby contributing to the inflammatory response and cell death (R. Fusco et al., 2020b). Several studies have indicated that the NLRP3 inflammasome participates in the pathogenesis of multiple neurological diseases, including traumatic brain injury (Irrera et al., 2017), Alzheimer's disease (Heneka et al., 2013), and intracerebral hemorrhage (Ren et al., 2018). Moreover, emerging research has revealed that inhibition of NLRP3 inflammasome activation can reduce inflammatory responses and attenuate EBI during SAH (Zhang et al., 2017; Zhou et al., 2018). Recently, RVD1 was reported to mitigate tissue damage by inhibiting NLRP3 activation (Li et al., 2017).

Based on the above-mentioned evidence, in the present study, we hypothesized that RVD1 protected the BBB by modulating A20 and NLRP3 to inhibit inflammation in a rat model of SAH.

## Materials and Methods

### Animals

A total of 279 male Sprague-Dawley rats (300–330 g) were purchased from the Laboratory Animal Center of the Southern Medical University (Guangzhou, China). Rats were kept in specific pathogen-free and humid conditions (12 h dark/light cycle). All experimental protocols complied with the requirements of the Southern Medical University Ethics Committee and the National Institutes of Health and Animal research guidelines.

### SAH Model

SAH model was constructed by endovascular perforation of rats as per previously reported methods (Schwartz et al., 2000). Briefly, rats were randomly assigned to sham, sham + vehicle, sham + RVD1, SAH, SAH + vehicle, SAH + RVD1, and SAH + RVD1+WRW4 groups and anesthetized with pentobarbital sodium (dosage: 40 mg/kg, intraperitoneal administration). Dissection and exposition of the left carotid artery and its branch vessels were performed. A sharpened 4–0 single-strand nylon thread was inserted into the left internal carotid artery from the external carotid artery cut off, and then the terminal bifurcation of the left internal carotid artery was punctured, inducing SAH. Sham group rats underwent a similar operation without vessel perforation. The suture was maintained in the vessel for approximately 10 s and then removed.

### Study Design

Three independent experiments were designed as described below. A total of 279 rats were used (Supplementary Figure S1B, C).

#### Experiment 1

To identify the time course of expression of RVD1, FPR2, and A20 after SAH as well as the cellular localization of FPR2 and A20, rats were randomly divided into the following groups: sham and SAH after 6, 12, 24, 48, and 72 h. The endogenous RVD1 concentration was measured with enzyme-linked immunosorbent assay (ELISA) kits (Omnimabs, Alhambra, CA, United States) according to the manufacturer's instructions. Western blot analysis was performed to determine the changes in levels of FPR2 and A20 proteins. The cellular localization of FPR2 and A20 was determined by immunofluorescence (IF) co-staining.

#### Experiment 2

To test the level of RVD1 in the brain tissue and to assess the neuroprotective effect after RVD1 injection in the rat model of SAH, three dosages were tested (0.1, 0.3, and 1.0 μg/kg). Rats were randomly distributed into five groups, namely sham, sham + vehicle [phosphate-buffered saline (PBS)], sham + RVD1, SAH + vehicle, SAH + RVD1 (0.1 μg/kg), SAH + RVD1 (0.3 μg/kg), and SAH + RVD1 (1.0 μg/kg). Neurological scores, Evans blue (EB) extravasation, and brain water content (BWC) were measured. Hematoxylin and eosin (H and E) staining was conducted at 24 h after SAH.

#### Experiment 3

To investigate the underlying mechanism of the protective role of RVD1, the optimal dose (0.3 μg/kg) of RVD1 was selected based on the results of experiment 2. The rats were randomly distributed into four experimental groups, namely sham, SAH + vehicle, SAH + RVD1, and SAH + RVD1+WRW4. Neurological scores, BWC, IF staining, immunohistochemical staining, terminal deoxynucleotidyl transferase-mediated dUTP nick-end labeling (TUNEL) staining, Fluoro-Jade C (FJC) staining, western blotting, and ELISA were performed at 24 or 72 h after SAH.

### Drug Administration

RVD1 was obtained from Cayman Chemical Company (Ann Arbor, MI, United States) and suspended in sterile PBS. RVD1 (0.1, 0.3, and 1.0 μg/kg) was injected via the femoral vein at 1 and 12 h after SAH induction. Vehicle (PBS) or WRW4 (66 μg/kg; Torcis, Bristol, United Kingdom), an FPR2 antagonist, was diluted in sterile PBS at 1 mg/ml and administered intracerebroventricularly 1 h before SAH induction (Deyama et al., 2017).

### Intracerebroventricular Drug Injection

Intracerebroventricular administration was performed as per previously reported methods (Liu et al., 2019b). Rats were anesthetized with 3% isoflurane and positioned in a stereotaxic apparatus. First, the rat skull was exposed and drilled at the following coordinates relative to the bregma: posterior 1.0 mm, lateral 1.5 mm, and 4.0 mm below endplate of the skull. A 25-μL Hamilton Syringe (Microliter 702, Hamilton Company, Reno, NV, United States) was inserted into the left lateral ventricle. The needle was held in place for 5 min after injection and pulled out slowly after another 5 min. Finally, the burr hole was closed with bone wax and the incision was sutured.

### Neurological Function Evaluation

Short-term neurological function was evaluated by an investigator who was blinded to the experimental protocol, using the modified Garcia scale and tests as per previously described methods (Garcia et al., 1995). The modified Garcia scale (maximum score of 18) assessed included touch of trunk, vibratory touch, spontaneous activity (0–3), spontaneous movements of four limbs (0–3), climbing capacity (1–3), and forelimbs stretching (0–3).

SAH grades were assessed blindly using the scoring system as per previously reported protocols (Sugawara et al., 2008). Rats with a grade <8 at 24 h after SAH were excluded from the study.

### BWC Analysis

Brain edema was assessed by determining the BWC at 24 h after SAH. The rats were euthanized; brain tissues were collected and divided into the left hemisphere (LH), right hemisphere (RH), cerebellum (CB), and brain stem (BS), and their wet weight was measured immediately. The samples were placed in an oven at 100°C for 48 h. The dry weight of the samples was then measured. BWC was calculated with the following formula: [(wet weight - dry weight)/wet weight] × 100%.

### Western Blotting

Western blotting was conducted as per methods described previously (Li et al., 2018). Briefly, equal amounts of total protein samples were resolved by SDS-PAGE and then blotted onto a nitrocellulose membrane. Membranes were blocked using 5% nonfat milk for 2 h at room temperature and then incubated with the following primary antibodies overnight at 4°C: anti-FPR2 (1:1,000, ab203129, Abcam, Cambridge, United Kingdom), matrix metallopeptidase (MMP)-9 (1:1000, cat#13667s, Cell Signaling Technology, Danvers, MA, United States), anti-zona occludens (ZO)-1 (1:1000, Santa Cruz Biotechnology, Dallas, TX, United States), anti-occludin (1:1000, ab167161, Abcam), anti-claudin-5 (1:1000, cat#AF5216, Affinity Biosciences, Cincinnati, OH, United States), anti-β-actin (1:1000, cat #4970s, Cell Signaling Technology), NLRP3 (1:600, cat#19771-1-AP, Proteintech, Rosemont, IL, United States), anti-caspase-1 (1:1000, cat#NBP1-45433, NOVUS Biologicals, Littleton, CO, United States), anti-NF-κB p65 (1:1000, cat#8242s, Cell Signaling Technology), phospho-NF-κBp65 (1:1000, cat#3033s, Cell Signaling Technology), IL-1β (1:1000, cat#AF5103, Affinity Biosciences), anti-Bax (1:1000, cat#AF0120, Affinity Biosciences), anti-Bcl-2 (1:1000, cat#AF6139, Affinity Biosciences), and anti-A20 (1:1000, cat#DF6850, Affinity Biosciences). Thereafter, the membrane was incubated with the appropriate secondary antibodies for 2 h at 26°C. Blot bands were detected with an image system (Bio-Rad, Hercules, CA, United States). The bands were quantified by densitometry analysis using the ImageJ version 1.5 software (NIH, Bethesda MD, United States).

### H and E Staining

H and E staining was performed as per previously described methods (Xie et al., 2015). Brain tissue was harvested and immobilized in 4% paraformaldehyde for 24–48 h. Brain tissue was then embedded in paraffin and coronal serial slices were obtained (thickness of 4 μm/slice). The slices were stained, and images were captured under a light microscope.

### IF Staining

Coronary paraffin-embedded brain sections (4 μm thick) were prepared as per previously described methods (Liu et al., 2019a). Sections were incubated with the following primary antibodies at 4°C overnight: rabbit anti-CD68 (1:200; Ab31630, Abcam), anti-myeloperoxidase (MPO) (1:50, ab90810, Abcam), intercellular cell adhesion molecule-1 (ICAM-1, 1:200, AF6088, Affinity Biosciences), anti-CD34 (1:200, AF5149, Affinity Biosciences), and anti-A20 (1:100, DF6850, Affinity Biosciences). After washing in PBS, the sections were incubated at room temperature for 1 h with the following corresponding secondary antibodies: Alexa Fluor 488 donkey anti-goat, Alexa 555 donkey anti-rabbit, Alexa Fluor 555 donkey anti-goat, Alexa Fluor 555 goat anti-mouse, and Alexa Fluor 488 donkey anti-rabbit (1:500; Invitrogen, Carlsbad, CA, United States). After washing three times in PBS and re-staining with 4′,6-diamidino-2-phenylindole, images were obtained using a fluorescence microscope (Leica-DMI8, Wetzlar, Germany).

### Immunohistochemical Staining

Rat brains were cut into coronal sections (4 μm thick) as per methods described earlier. Endogenous peroxide was inactivated by adding 3% H_2_O_2_ for 15 min at room temperature. The sections were blocked with 5% bovine serum albumin for 15 min and incubated overnight with the following primary antibodies at 4°C: anti-MMP9 (1:300, cat#13667s, Cell Signaling Technology), anti-Iba1 (1:200; cat#Ab5076, Abcam), and anti-caspase1p20/p10 (1:200, cat# 22915-1-AP, Proteintech). Brain slices were washed with PBS and incubated (20 min) with biotinylated goat anti-rabbit IgG followed by streptavidin-horseradish peroxidase (15 min). Peroxidase activities were detected by using 3,3-diaminobenzidine. Images were obtained under a light microscope (Leia-DM2500).

### TUNEL Staining

The slices were prepared as per methods described above. Briefly, the slices were treated with proteinase K at 37°C for 25 min and with 0.1% Triton for 20 min at 37°C. Then, TUNEL staining was conducted following the manufacturer's instructions (Roche, Basel, Switzerland). Stained positive cells were calculated in each field of the slice. Six areas of each section were randomly selected and recorded under a microscope, and then calculated using the ImageJ version 1.5 software.

### Fluoro-Jade C (FJC) Staining

FJC staining to detect neuronal degeneration was performed as per methods reported previously (Liu et al., 2019a). Briefly, brain sections were immersed in 1% NaOH/80% ethanol and sequentially rinsed in 70% ethanol for 2 min, and then immersed in 0.06% potassium permanganate solution for 10 min. Next, sections were placed in a 0.0001% solution of FJC (Millipore, Darmstadt, Germany), which was solubilized in 0.1% acetic acid for 30 min. After rinsing the sections with distilled water, they were dried at 50°C for 10 min. Sections were then imaged by fluorescence microscopy (Leica-DMI8).

### ELISA

At 24 h after SAH, the rat brain tissue homogenates were obtained and centrifuged for 20 min (4°C, 12,000 × *g*). The supernatant was harvested and kept at −80°C until use. IL-18, IL-10, and TNF-α levels in the brain tissue lysates were detected with ELISA kits (R&D Systems, Minneapolis, MN, United States) according to the manufacturer's instructions. The samples were detected by spectrometry using a microplate reader. Cytokine concentrations were determined by constructing a standard curve.

### Statistical Analysis

All data are expressed as mean ± standard deviation. Statistical analyses were performed using the SPSS version 19.0 software (SPSS, Inc., Chicago, IL, United States). The Student's *t-*test or Mann-Whitney U test were used to compare two groups after testing the data for normality. Multiple groups were compared by using one-way or two-way analysis of variance with the Bonferroni test. A *p*-value (*p*) < 0.05 was considered statistically significant.

## Results

### SAH Grades and Mortality

**FIGURE 1:**
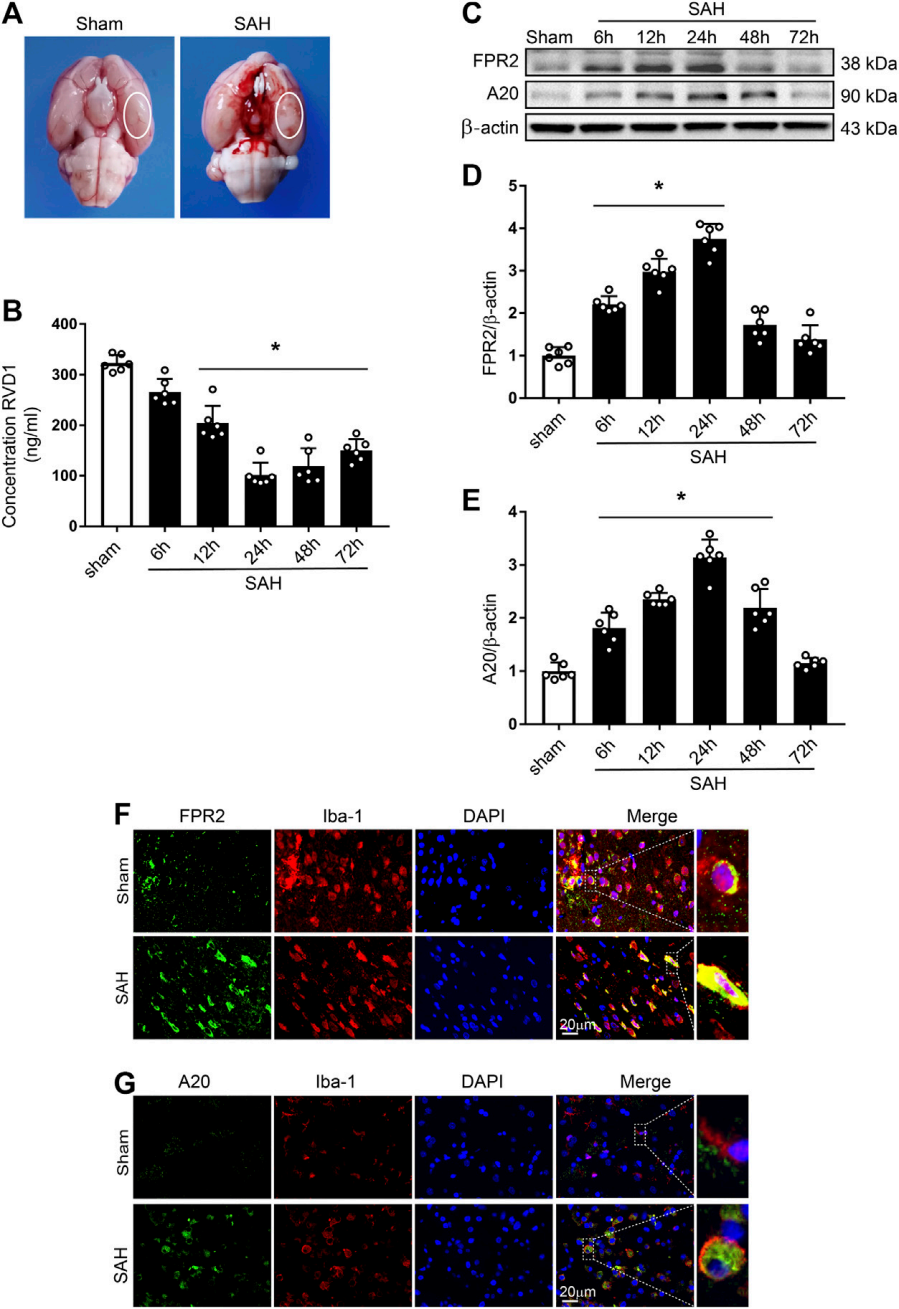
Time course of expression of endogenous RVD1, FPR2, and A20 after subarachnoid hemorrhage (SAH). **(A)** Brain images of sham and SAH rats. Schematic illustration of the optimal brain region for staining with a white oval frame. **(B)** ELISA indicating RVD1 expression after SAH. **(C)** Representative western blot bands of FPR2 and A20 **(D, E)**. The intensity of each protein band was quantified and normalized with β-actin. **(F)** Representative images of double immunofluorescence staining show that FPR2 and A20 (green) are highly expressed in microglia (Iba-1, red). Scale bar = 20 μm; *p < 0.05 vs. sham group, n = 6 per group.

In rats, SAH was induced primarily around the circle of Willis and ventral brainstem (Figure 1A). There was no significant difference in the mean SAH grade between all SAH groups. No rats died in the sham (0/36 rats), sham + vehicle (0/6 rats), and sham + RVD1 (0/6 rats) groups. The mortality rates were 21.73% (10/46 rats) and 22.22% (12/54 rats) in the SAH and SAH + vehicle groups, respectively. The mortality rates were 18.18% (4/22 rats), 10.67% (5/47 rats), and 10.00% (2/20 rats) in the SAH + RVD1 (0.1, 0.3, and 1.0 μg/kg) groups, respectively. The SAH + RVD1+WRW4 group mortality rate was 21.73% (5/23 rats). Eleven rats were excluded from the study because they showed only low grade (<8) SAH at 24 h.

### Time-Course of Endogenous RVD1, FPR2, and A20 Expression After SAH

Levels of endogenous RVD1 in the left hemisphere were detected by ELISA. The data showed the commencement of a gradual decline of RVD1 levels at 6 h after SAH, with the most significant decrease at 24 h (*p* < 0.05; Figure 1B). Endogenous protein expression of FPR2 was evaluated by western blotting and IF staining. Compared to the sham group, expression of both FPR2 and A20 was upregulated at 6 h after SAH and peaked at 24 h (Figure 1C–E). Double immunofluorescence staining showed that both FPR2 and A20 were highly expressed in the microglia (Figure 1F,G), meanwhile A20 was expressed at low levels in the astrocytes and neurons after SAH (Supplementary Figure S2B).

### Administration of RVD1 Reduces Brain Edema and Improves Neurological Functions at 24 h after SAH

**FIGURE 2:**
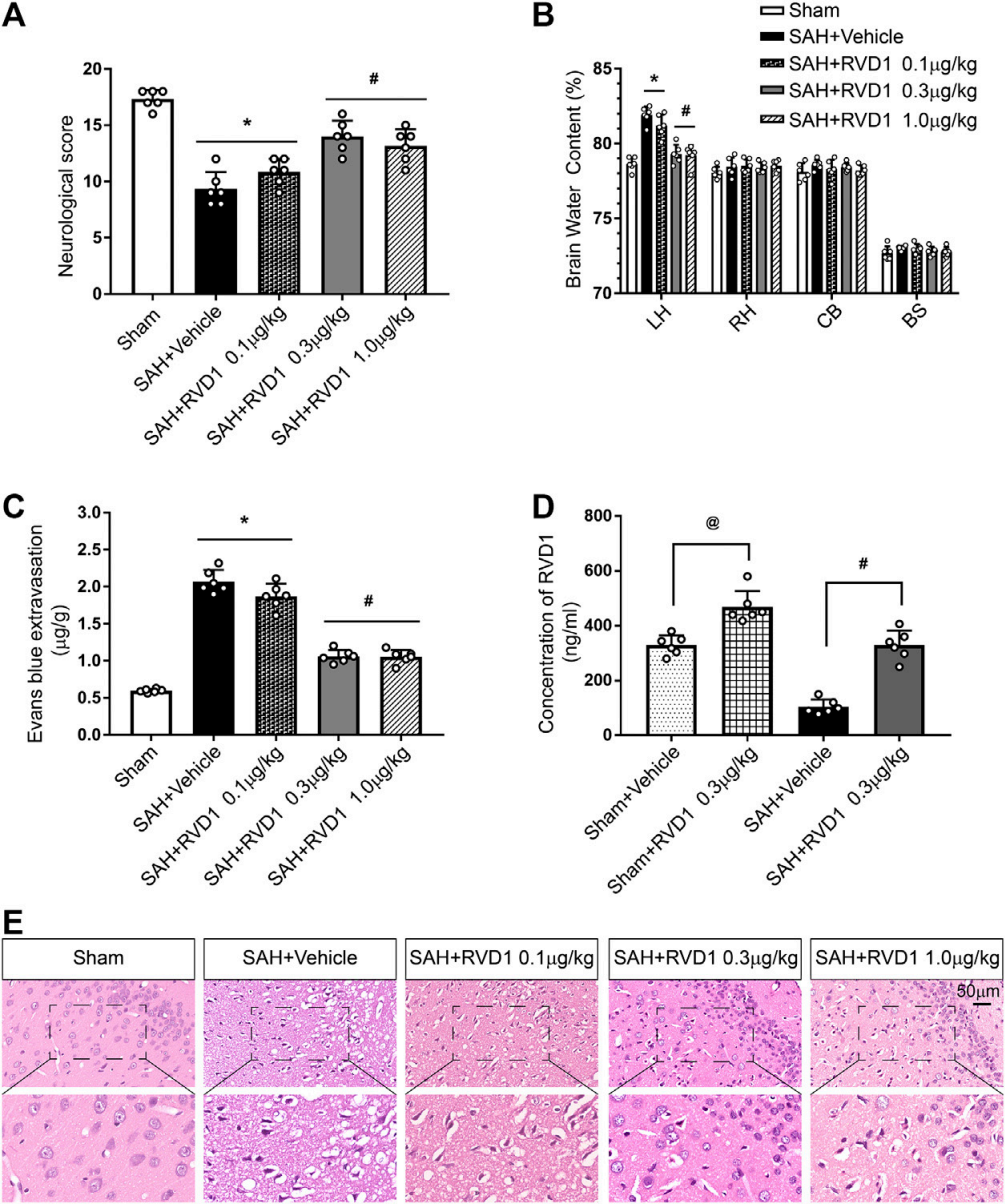
Effects of three dosage levels of RVD1 administration on SAH in rats. **(A)** Neurological score, **(B)** Brain water content, and **(C)** Evans blue extravasation in each group. **(D)** Changes in RVD1 concentration in the brain tissue before and after RVD1 administration. **(E)** Representative images of H and E staining of the ipsilateral temporal cortex in different groups. Scale bar = 50 μm; ^@^ *p* < 0.05 vs. sham + vehicle group, *p < 0.05 vs. sham group, ^#^ *p* < 0.05 vs. SAH + vehicle group; n = 6 per group.

To investigate the effect of exogenous RVD1 on EBI after SAH, three doses of RVD1 (0.1, 0.3, and 1.0 μg/kg) were administered after SAH. As shown in Figure 2A–C, there were significantly lower neurological scores, higher levels of EB exfiltration, and BWC in the SAH + vehicle group than those in the sham group (*p* < 0.05). In contrast, after RVD1 administration at doses of 0.3 and 1.0 μg/kg, EB exfiltration and BWC were significantly decreased, and neurological deficits were improved at 24 h after SAH. The H&E-stained slices of the cortex showed that RVD1 administration significantly ameliorated histological damage at doses of 0.3 and 1.0 μg/kg compared to that in the SAH + vehicle group (*p* < 0.05; Figure 2E). The level of RVD1 in the rat brain tissue was significantly increased in both the sham and SAH groups at 24 h after administration of 0.3 μg/kg RVD1 (*p* < 0.05; Figure 2D and Supplementary Figure S2A). Based on the above-mentioned results, we selected 0.3 μg/kg as the optimal dose in subsequent experiments.

### RVD1 Inhibition of NLRP3 Inflammasome and Microglia Activation Reduces Inflammation after SAH

**FIGURE 3:**
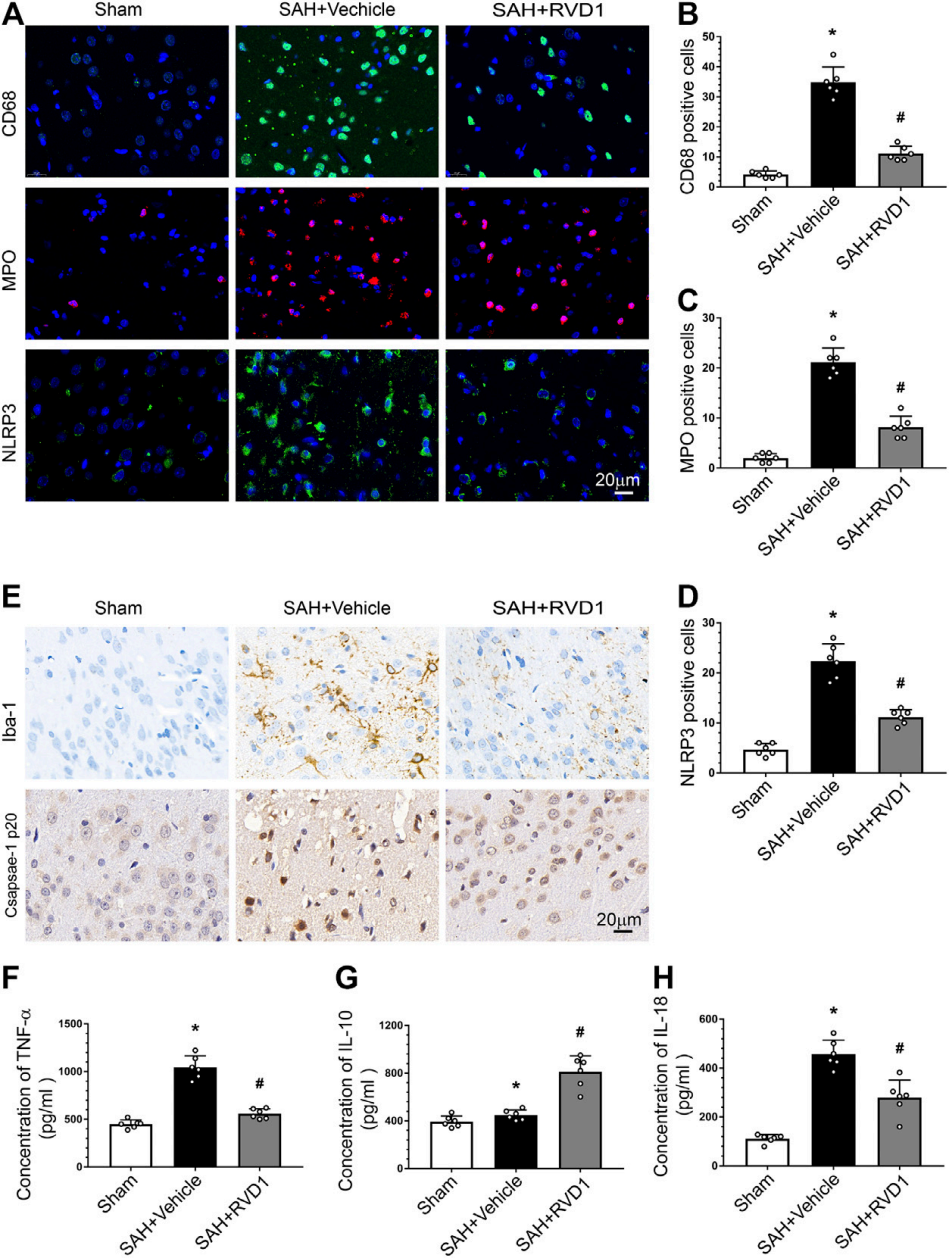
Effects of RVD1 administration on inflammation after SAH. Representative images of **(A)** immunofluorescence staining of CD68, MPO, and NLRP3, and **(E)** immunohistochemistry for Iba-1 (microglia marker) and caspase1 p20. Quantitative analysis of **(B)** CD68, **(C)** MPO, and **(D)** NLRP3 immunofluorescence staining per field. ELISA quantification of **(F)** TNF-α, **(G)** IL-18, and **(H)** IL-10 secretion. Scale bar = 20 μm; *p < 0.05 vs. sham, ^#^ *p* < 0.05 vs. SAH + vehicle group, and ^&^ *p* < 0.05 vs. SAH + RVD1 group; n = 6 per group.

Activation of immune cells and the release of inflammatory factors are hallmarks of neuroinflammation. We performed IF staining for CD68 and MPO to identify activated microglia and neutrophils, respectively. The result showed that the numbers of CD68^−^, MPO-, and NLRP3-positive cells were markedly increased at 24 h after SAH. RVD1 treatment significantly reduced the numbers of CD68^−^, MPO-, and NLRP3-positive cells in the ipsilateral cortex compared to those in the SAH + vehicle group (*p* < 0.05; Figure 3A–D). Immunohistochemical staining revealed that Iba-1 and caspase-1p20 were also significantly increased at 24 h after SAH, whereas RVD1 administration reversed these increases (Figure 3E). SAH increased the expression of the proinflammatory cytokines IL-18 and TNF-α. In contrast, RVD1 treatment downregulated the expression of IL-18 and TNF-α but upregulated that of the anti-inflammatory factor IL-10 (Figures 3F–H).

### RVD1 Decreases the Expression of MMP-9, Upregulates Tight Junction Proteins, and Attenuates BBB Disruption at 24 h after SAH

**FIGURE 4:**
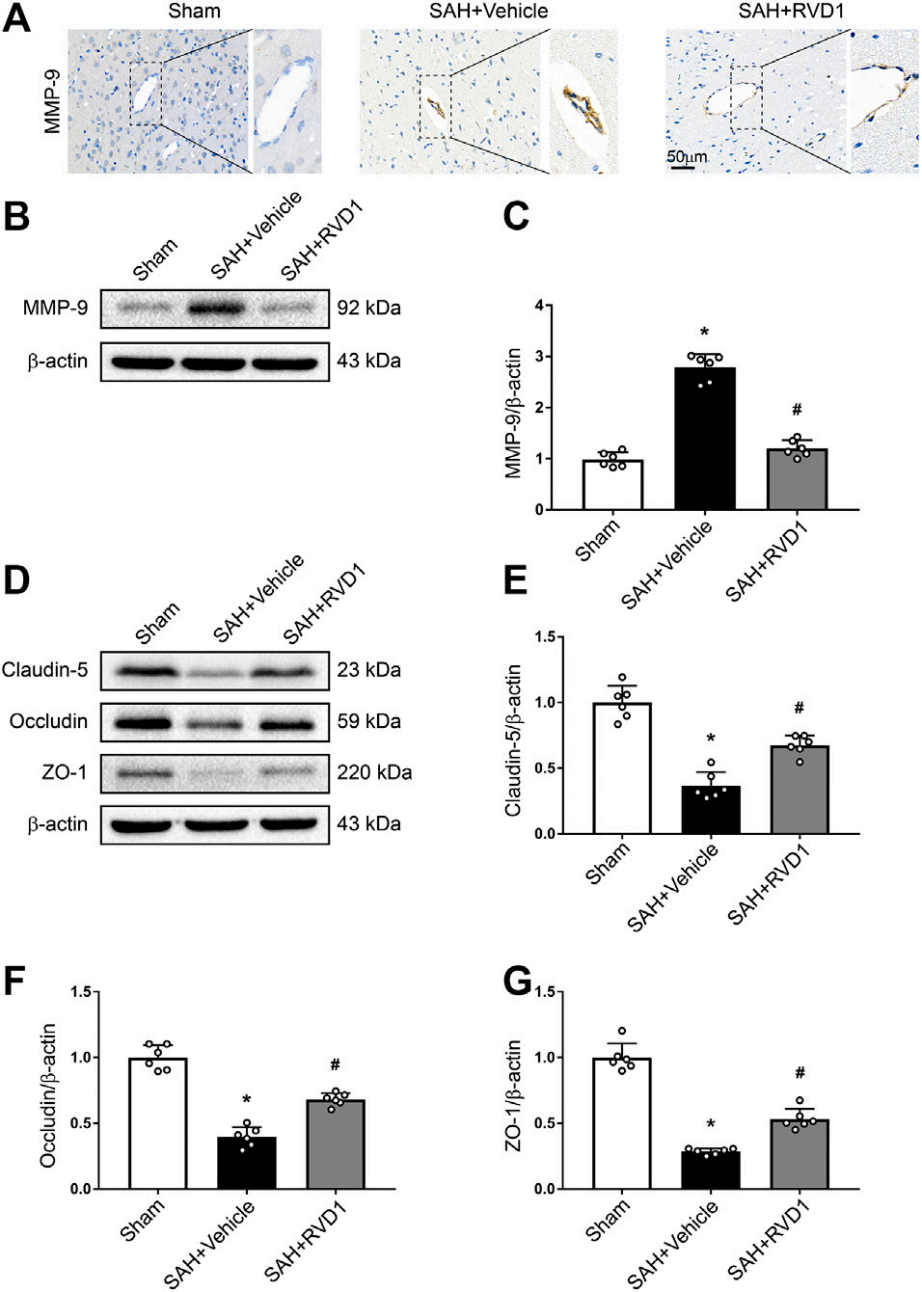
The effect of RVD1 on maintenance of blood-brain barrier integrity after SAH. **(A)** Representative images of ICH staining for MMP-9 in the cerebral cortex vascular tissues after SAH. **(B)** Representative western blot bands and quantitative analysis of the expression of **(C)** MMP-9, **(D)** tight junction-related proteins, **(E)** claudin-5, **(F)** occludin, and **(G)** ZO-1 in the ipsilateral cortex at 24 h post-SAH. Scale bar = 50 μm; *p < 0.05 vs. sham group, ^#^ *p* < 0.05 vs. SAH + vehicle group; n = 6 per group.

MMP-9 was highly expressed in the collapsed vessels at 24 h after SAH. MMP-9 expression in the vascular tissues was significantly suppressed after RVD1 injection, and vascular morphology was restored in the cortex (Figure 4A). Furthermore, RVD1 upregulated tight junction protein expression (occludin-5, claudin, and ZO-1) and reduced MMP-9 compared to the corresponding expression levels in the SAH + vehicle group (Figure 4B–G).

### RVD1 Suppresses Neutrophil Migration at 24 h after SAH and Promotes Neutrophil Apoptosis at 72 h after SAH

**FIGURE 5:**
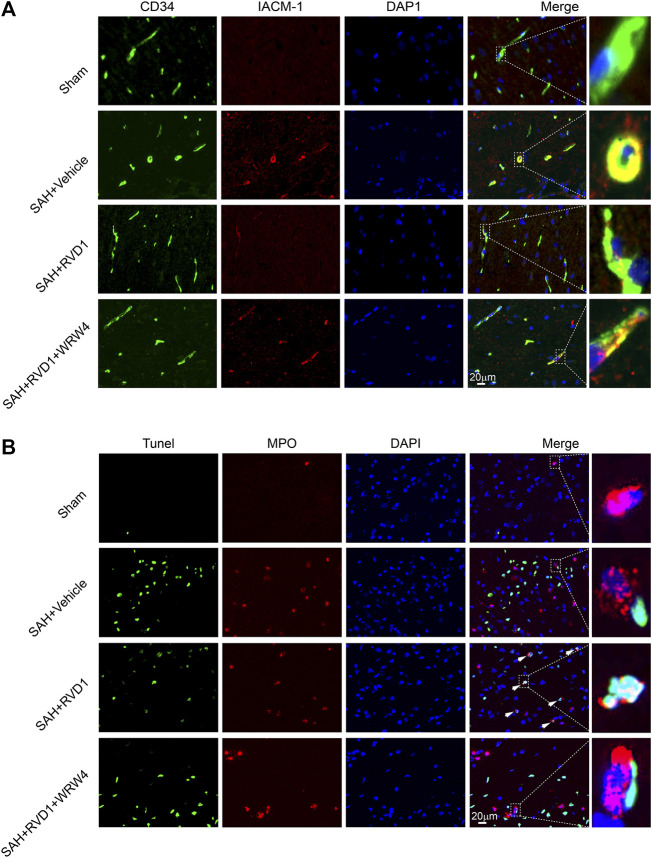
The effect of RVD1 and WRW4 on neutrophil infiltration and apoptosis after SAH. **(A)** Representative immunofluorescence co-localization images of ICAM-1 (red) and CD34 (green) in different groups. **(B)** Co-localization of TUNEL (green) and MPO (neutrophil marker, red) immunofluorescence staining at 72 h after SAH. TUNEL-positive neutrophils (white arrowheads) in the RVD1 group. Scale bar = 20 μm; n = 6 per group.

Neutrophils are key immune cells that mediate the inflammatory response. To investigate the regulatory effect of RVD1 on neutrophils after SAH, we detected ICAM-1 expression in microvascular endothelial cells and neutrophil apoptosis in the cortex by IF staining (Figure 5A,B). ICAM-1 expression was significantly increased in the microvascular endothelium at 24 h after SAH. RVD1 treatment suppressed the expression of ICAM-1 (Figure 5A). Interestingly, we found that RVD1 administration increased neutrophil apoptosis in the cortex at 72 h after SAH (Figure 5B). However, this modulatory effect of RVD1 on neutrophils was reversed after WRW4 injection.

### RVD1 Reduces Apoptosis and Neuronal Degeneration after SAH, and WRW4 Reverses the Neuroprotective Effects of RVD1

**FIGURE 6:**
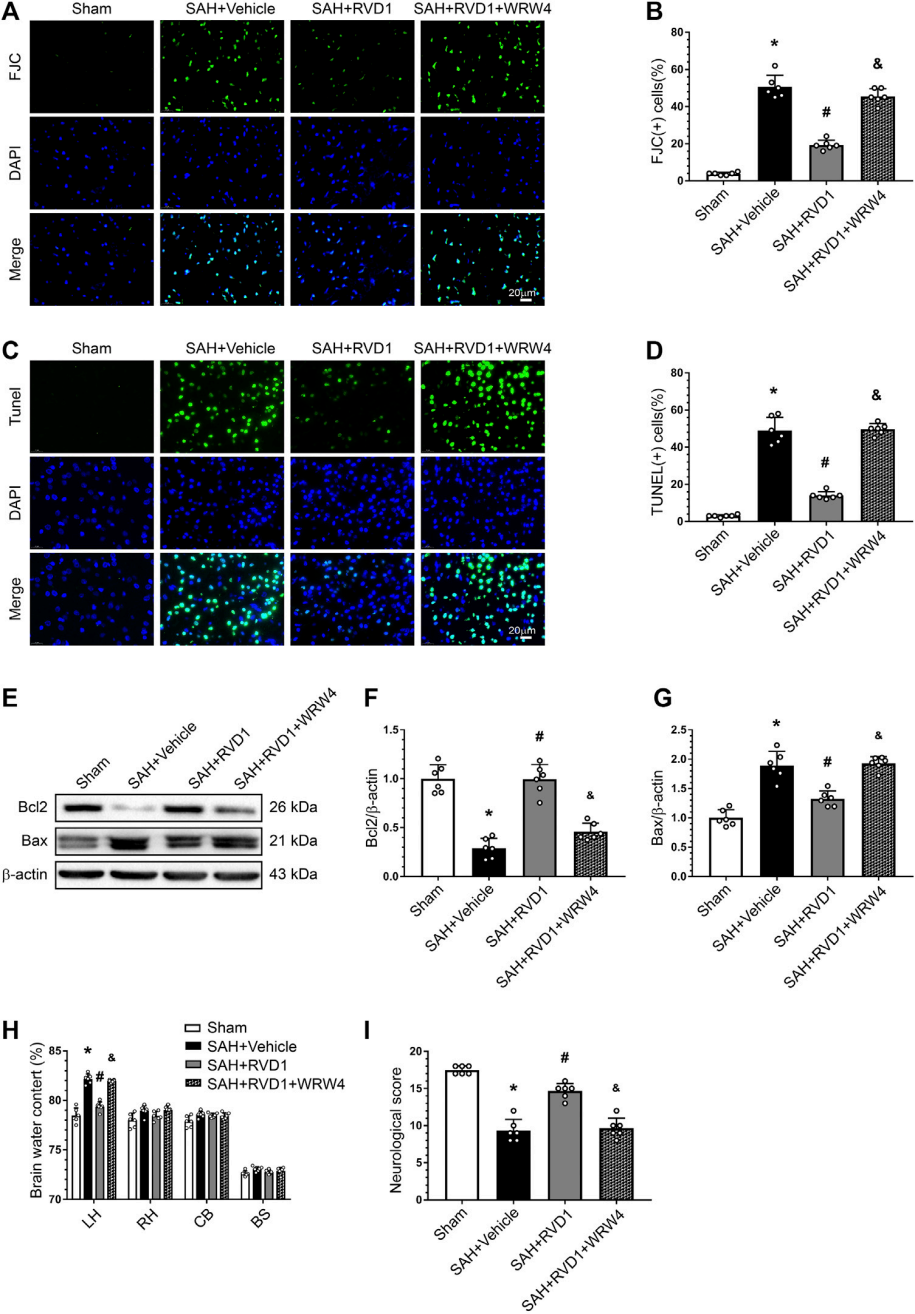
Effects of RVD1 and WRW4 on neuronal cells after SAH. **(A, C)** Representative microfluorescence images and **(B, D)** quantitative analysis of FJC- and TUNEL-positive cells. **(E)** Representative western blot bands and **(F, G)** quantitative analysis of Bcl2 and Bax expression. **(H)** Brain water content. **(I)** Neurological score in different groups. Scale bar = 20 μm; *p < 0.05 vs. sham group, ^#^ *p* < 0.05 vs. SAH + vehicle group, ^&^ *p* < 0.05 vs. RVD1 group; n = 6 per group.

FJC- and TUNEL-positive cells were markedly increased in the cerebral cortex after SAH. Consistently, the Bcl-2 protein level decreased dramatically, whereas Bax protein was increased. RVD1 administration significantly reduced neuronal apoptosis and neuronal degeneration at 24 h after SAH. However, blocking of FPR2 with WRW4 significantly attenuated the effect of RVD1 against apoptosis and neurodegeneration (*p* < 0.05; Figure 6A–G). WRW4 also exacerbated neurological deficits and brain edema (*p* < 0.05; Figure 6H,I).

### RVD1 Attenuates Inflammation via the FPR2/A20 Pathway and Inhibits the NLRP3 Inflammasome to Maintain BBB Integrity

**FIGURE 7:**
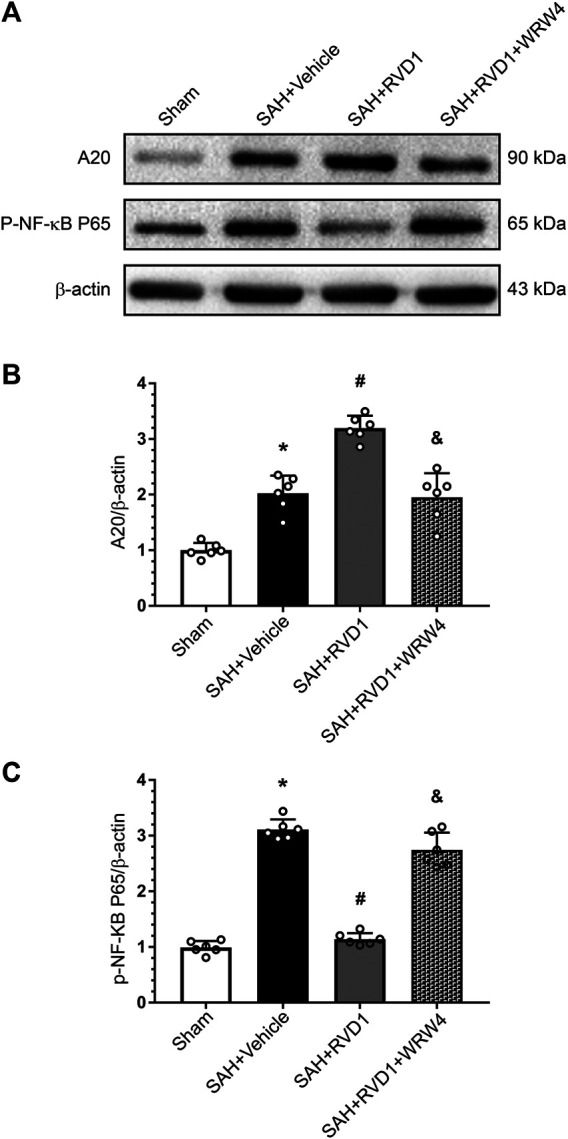
The effect of RVD1 and WRW4 on the A20/NF-κB pathway after SAH. **(A)** Representative western blot images and **(B**, **C)** quantitative analysis of A20 and NF-κB expression. *p < 0.05 vs. sham, ^#^ *p* < 0.05 vs. SAH + vehicle group, ^&^ *p* < 0.05 vs. RVD1 group; n = 6 per group.

**FIGURE 8:**
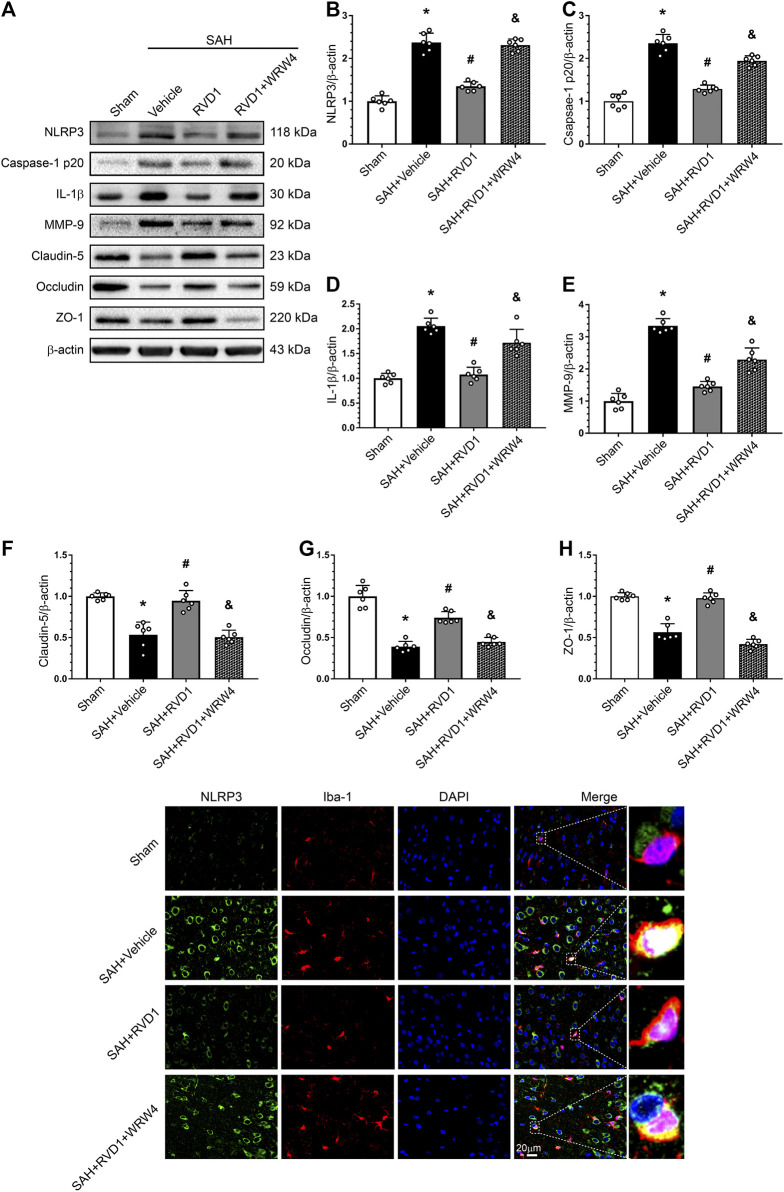
Effects of RVD1 and WRW4 on NLRP3/caspase-1/IL-1β and the blood-brain barrier after SAH. **(A)** Representative western blot bands and **(B–H)** quantitative analysis of NLRP3, caspase-1p20, IL-1β, MMP-9, claudin-5, occludin, and ZO-1. **(I)** Representative double immunofluorescence staining images of NLRP3 (green) and Iba-1 (red). *p < 0.05 vs. sham group, ^#^ *p* < 0.05 vs. SAH + vehicle group, ^&^ *p* < 0.05 vs. SAH + RVD1 group; n = 6 per group.

Western blot data showed that RVD1 treatment markedly increased A20, occludin-5, claudin, and ZO-1 expression levels. In contrast, the expression levels of p-NF-κB p65, NLRP3, cleaved caspase-1, IL-Iβ, and MMP-9 were significantly down-regulated compared to those in the SAH + vehicle group (Figures 7A–C, Figure 8A–I). Double IF staining results confirmed that RVD1 treatment significantly reduced NLRP3 expression in the microglia compared to that in the SAH + vehicle group (Figure 8J). By blocking FPR2 with WRW4, the effects of RVD1 were abolished at 24 h after SAH (*p* < 0.05; Figures 7A–C, 8A–J).

## Discussion

In this study, we evaluated the role and explored the potential mechanisms of RVD1 in EBI after SAH. The results revealed that endogenous expression of RVD1 was decreased, whereas FPR2 and A20 expression levels were increased in a time-dependent manner and peaked at 24 h, after SAH. FPR2 and A20 were colocalized in the microglia. We also showed that intravenous RVD1 treatment (0.3 or 1.0 μg/kg) could improve neurofunction, reduce brain edema, and alleviate BBB disruption after SAH in rats. RVD1 treatment down-regulated NLRP3 expression in microglia and ICAM-1 expression in vascular endothelial cells, inhibited microglial activation and neutrophil infiltration, significantly increased the expression of the anti-inflammatory molecule A20, promoted neutrophil apoptosis, and ameliorated neuronal degeneration. WRW4, an FPR2 inhibitor, abolished the effects of RVD1 neuroprotection and anti-inflammation. Overall, our data indicate that RVD1 improves neurofunction, mitigates inflammation, protects the BBB partially via the FPR2/A20 pathway, and deactivates the NLRP3 inflammasome in EBI after SAH.

Inflammation triggers BBB disruption, cell death, and oxidative stress in EBI after SAH (Geraghty et al., 2019). Microglia and neutrophils are key immune cells involved in the inflammatory response in CNS inflammation-related diseases, such as hypoxic-ischemic brain injury and SAH (Z. Xu et al., 2019b; Yao and Kuan, 2020). FPR2, a member of the G protein-coupled receptor family, plays an important role in host defense and cellular debris clearance (Weiss and Kretschmer, 2018). FPR2 is abundantly expressed in neutrophils and can serve as a target for regulation of neutrophil apoptosis and enhancement of the resolution of inflammation (Filep and El, 2009; Dahlgren et al., 2016). Notably, RVD1 exhibited anti-inflammatory effects and accelerated inflammation resolution by binding to FPR2 with high affinity (Basil and Levy, 2016; Isopi et al., 2020). We observed that FPR2 co-localized with microglia, which was consistent with findings of a previous study (Guo et al., 2016). This suggests that microglia and neutrophils can be used as targets of RVD1 for SAH treatment. Our results showed that RVD1 suppressed microglial activation and limited neutrophil infiltration while inhibiting the release of inflammatory cytokines TNF-α, IL-18, and IL-1β, thereby increasing anti-inflammatory cytokine IL-10. These results agree with those of a former study (Liu et al., 2019a).

Previously, the resolution of inflammation was considered a passive procedure (Tabas and Glass, 2013). However, emerging studies have revealed that pro-resolving lipid mediators mediate the active resolution of inflammation, although the underlying mechanisms remain poorly understood (Serhan, 2014). In this study, neutrophils were labeled with MPO, and the number of TUNEL-positive MPO cells significantly increased and exhibited nuclear collapse in immunofluorescence staining. The results indicated that RVD1 not only limited neutrophil infiltration, but also promoted neutrophil apoptosis after SAH. Furthermore, we found that high expression of ICAM-1 in vascular endothelial cells after SAH was suppressed by RVD1 treatment. Inhibition of ICAM-1, a key molecule regulating neutrophil adhesion and infiltration, can reduce neutrophil adhesion and infiltration (DiStasi and Ley, 2009). This reveals that RVD1 restriction of neutrophil infiltration may be mediated through inhibition of ICAM-1. While the potential mechanism by which RVD1 promoted neutrophil apoptosis was not explored in this study, this mechanism might be related to the activation of the FPR2/JNK/caspase3 pathway in neutrophils (Cooray et al., 2013).

Zinc finger protein A20 is encoded by the TNFAIP3 gene. As a ubiquitin-editing enzyme, A20 regulates intracellular signaling from pattern recognition receptors to NF-κB via ubiquitin modification, which inhibits the activation of NF-κB and release of IL-1β. A20 plays a crucial role in anti-inflammation and improvement of cell survival (Priem et al., 2020). Previous studies showed that overexpression of A20 reduced acute inflammation in intracerebral hemorrhage and ischemic stroke (Zhan et al., 2016; Perga et al., 2020). We observed high expression of A20 in microglia, but low expression in astrocytes and neurons, and its expression peaked 24 h after SAH. While the cellular localization of A20 in SAH has not been previously reported, A20 is predominantly expressed in neurons and microglia in a mouse model of intracerebral hemorrhage, with its expression peaking at 3 days after intracerebral hemorrhage (Meng et al., 2017). Expression of A20 may vary due to differences between the models; in our rat SAH model, hemorrhage was induced by puncturing large blood vessels on the brain surface, whereas in the ICH model, hemorrhage was induced by injection of autologous blood slowly into the brain parenchyma. Therefore, the inflammatory response initiation in SAH may be more rapid, intense, and with more immune cell involvement than that in ICH. We observed upregulation of A20 expression after RVD1 treatment, which was abolished after WRW4 blocked the FPR2 receptor, suggesting that A20 was a downstream molecule of FPR2.

Tight junction proteins (ZO-1, occludin, and claudin-5) are key components of the BBB that maintain brain microenvironment homeostasis (Lochhead et al., 2020). MMP-9 degrades tight junction proteins in a variety of neurological diseases, including ischemic stroke, traumatic brain injury, and SAH (Egashira et al., 2015; Lu et al., 2018; Yang et al., 2020). A previous study showed that IL-1β increased MMP-9 expression, triggered ZO-1 degradation, and opened the BBB after SAH (Sozen et al., 2009). Furthermore, MMP-9 activation accelerated leukocyte crossing of the BBB (Song et al., 2015). Upregulation of A20 also protected the BBB integrity by inhibiting NF-κB activation (Han et al., 2016). A previous study suggested that RVD1 ameliorated LPS-induced BBB disruption by upregulating HO-1 (Xie et al., 2013). Our data showed that RVD1 reduced EB extravasation and IL-1β and MMP-9 expression, and upregulated A20 and tight junction protein after SAH. These results suggest that RVD1 reduces BBB disruption in association with upregulation of A20.

NLRP3/caspase 1/IL-1β activation induces pyroptosis, a novel form of programmed cell death (Martinon et al., 2002). A growing body of evidence has demonstrated that NLRP3 plays an important inflammatory role in EBI after SAH. It has been reported that a specialized pro-resolving lipid mediator negatively regulates the NLRP3 inflammasome via the NF-κB pathway (Lopategi et al., 2019; Wang et al., 2020). In the present study, NLRP3 was expressed in the microglia and was significantly upregulated at 24 h after SAH. RVD1 significantly decreased the expression of phosphorylated NF-κB and NLRP3, caspase-1, and IL-1β after SAH, However, WRW4 abolishes this effect. A previous study has suggested that A20 is an upstream molecule that inhibits NLRP3 activation (Duong et al., 2015; Voet et al., 2018). In the present study, RVD1 upregulation of A20 may have been involved in regulation of NLRP3 expression.

Pro-inflammatory factors act on death receptors to trigger a caspase-dependent pathway that cleaves Bcl-2 and activates pro-apoptotic proteins, including Bax, which are involved in mediating neuronal apoptosis (Duris et al., 2018). We found that RVD1 treatment promoted Bcl2 expression, inhibited Bax, and reduced neuronal apoptosis. As our results show that RVD1 protects the BBB, inhibits immune cell infiltration and activation, and significantly decreases inflammatory factors, RVD1 may exert its anti-apoptotic effect by reducing inflammation. RVD1 has also been shown to protect astrocytic mitochondria and reduce reactive oxygen species, thereby reducing neuronal apoptosis in mice of traumatic brain injury (Ren et al., 2020). The specific anti-apoptotic mechanism of RVD1 will be addressed in future studies.

There are several limitations to this study. We cannot exclude the fact that other mechanisms are involved in RVD1-mediated alleviation of EBI after SAH. First, regulation of neuroinflammation typically involves a complex signaling network, such as microglial polarization, which is important in neuroinflammation after SAH (Li et al., 2018). Here, we demonstrated that RVD1 inhibited SAH-induced microglial activation but its effect on the microglial polarization was not determined. Second, cerebral vasospasm with delayed cerebral ischemia remains an important factor affecting the poor prognosis after SAH (Neifert et al., 2020). More recently, it was shown that RVD1 relaxed vascular smooth muscles and attenuated vasoconstriction, as well as enhanced perfusion recovery in ischemia injury (Sansbury et al., 2020). Therefore, further studies are warranted to determine the mechanisms of RVD1 in SAH.

In conclusion, we demonstrated that RVD1 attenuated inflammation-mediated BBB disruption and improved neurological deficits in the rat SAH model. This protective effort of RVD1 was, to some extent, partially mediated by the FPR2/A20 pathway and inhibition of the NLRP3 inflammasome activation. Therefore, RVD1 may serve as a potential new drug and FPR2 as a novel pharmacological target for the treatment of SAH.

## Data Availability

The original contributions presented in the study are included in the article/Supplementary Material, further inquiries can be directed to the corresponding author.

## Abbreviations

BBB, blood-brain barrier; EBI, early brain injury; FPR2, formyl peptide receptor 2; MPO, myeloperoxidase; RVD1, resolvin D1; SAH, subarachnoid hemorrhage; TUNEL, terminal deoxynucleotidyl transferase-mediated uridine triphosphate nick-end labeling; WRW4, Trp-Arg-Trp-Trp-Trp-Trp-NH2.
