# Supplementary figures and images for: Resolvin D1 ameliorates Inflammation-Mediated Blood-Brain Barrier Disruption After Subarachnoid Hemorrhage in rats by Modulating A20 and NLRP3 Inflammasome
Source: Front Pharmacol. 2021 Feb 3;11:610734. doi: 10.3389/fphar.2020.610734 (PMC7957930; doi:10.3389/fphar.2020.610734)

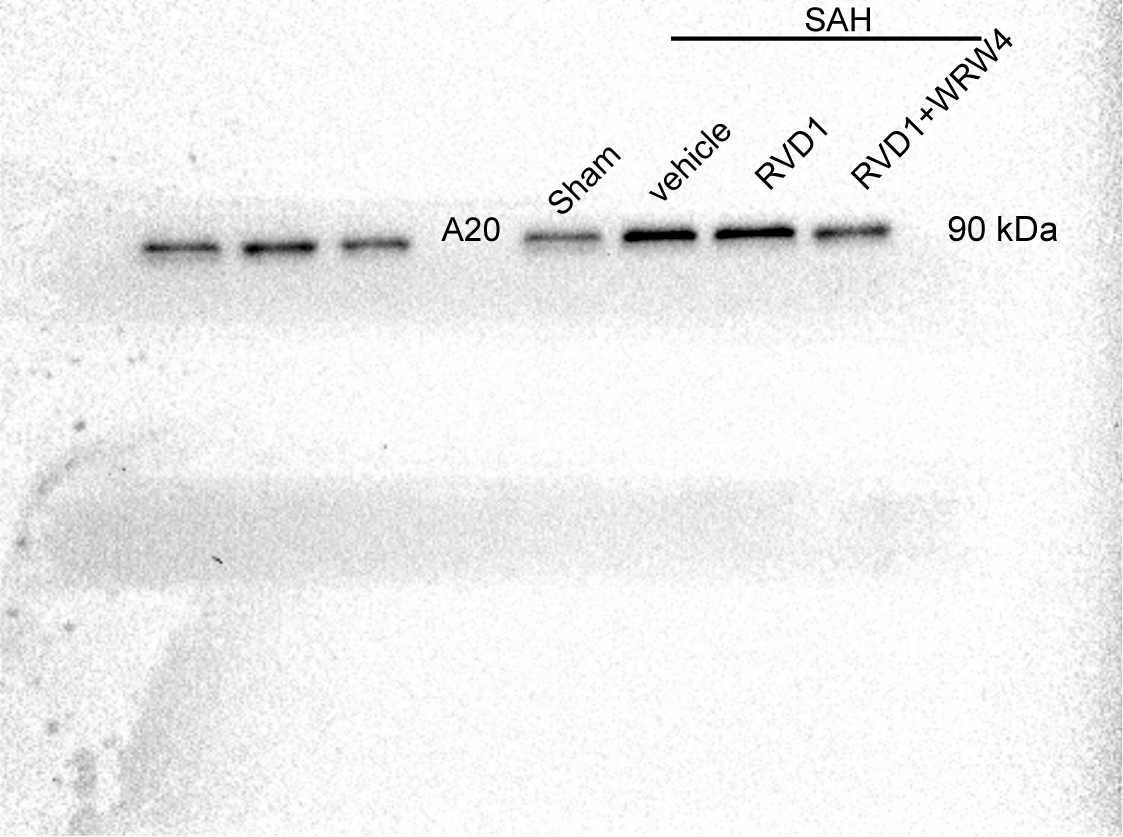

Supplement: Supplementary file 1 [file datasheet1.zip › A20-original images/A20/A20-1-Administrator 2019-12-19 09 ╩▒ 54 ╖╓_Exposure_198.0sec.tif]

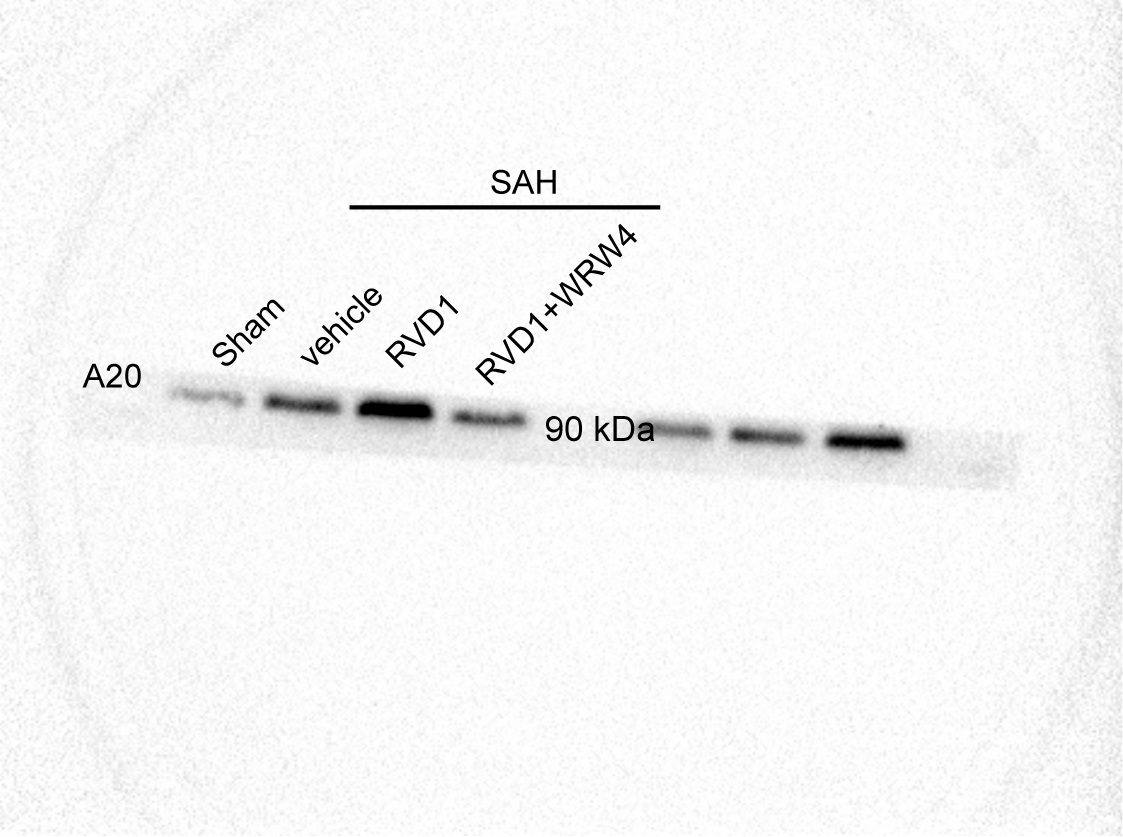

Supplement: Supplementary file 1 [file datasheet1.zip › A20-original images/A20/A20-2-Administrator 2020-06-16 23 ╩▒ 46 ╖╓_Exposure_53.5sec.tif]

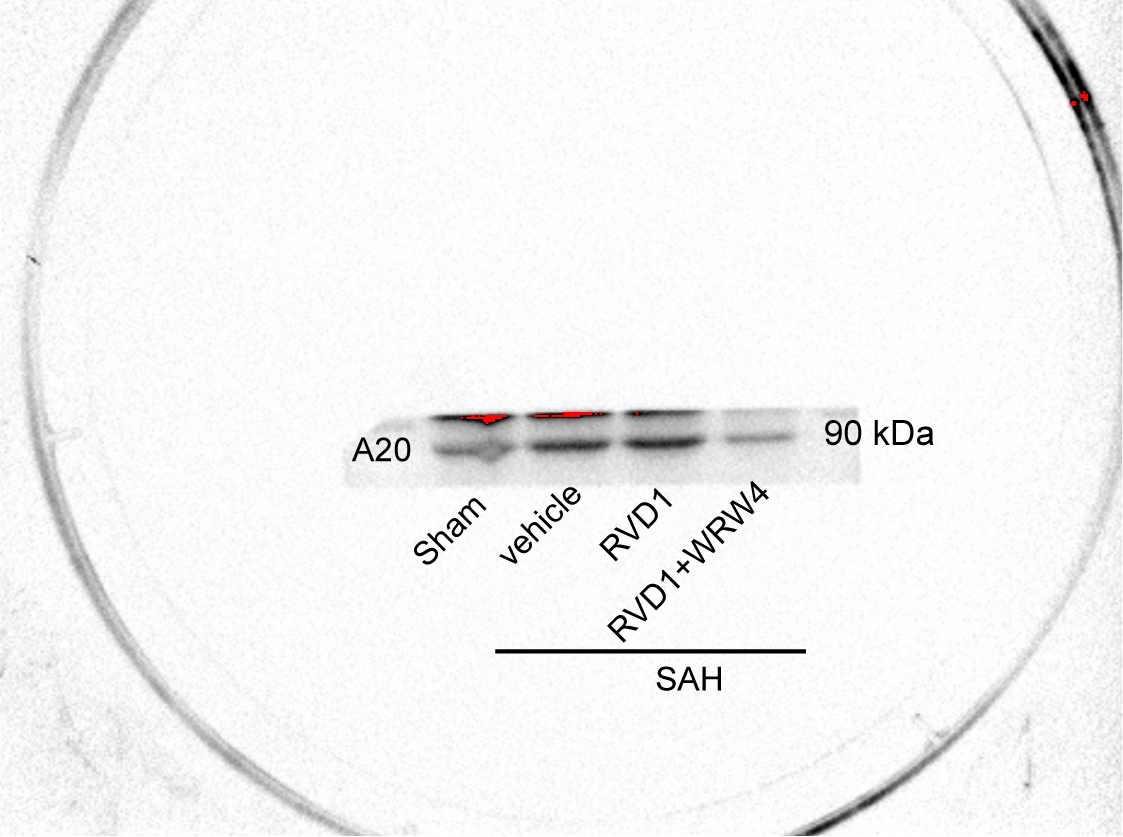

Supplement: Supplementary file 1 [file datasheet1.zip › A20-original images/A20/A20-3-Administrator 2020-06-16 23 ╩▒ 59 ╖╓_Exposure_105.5sec.tif]

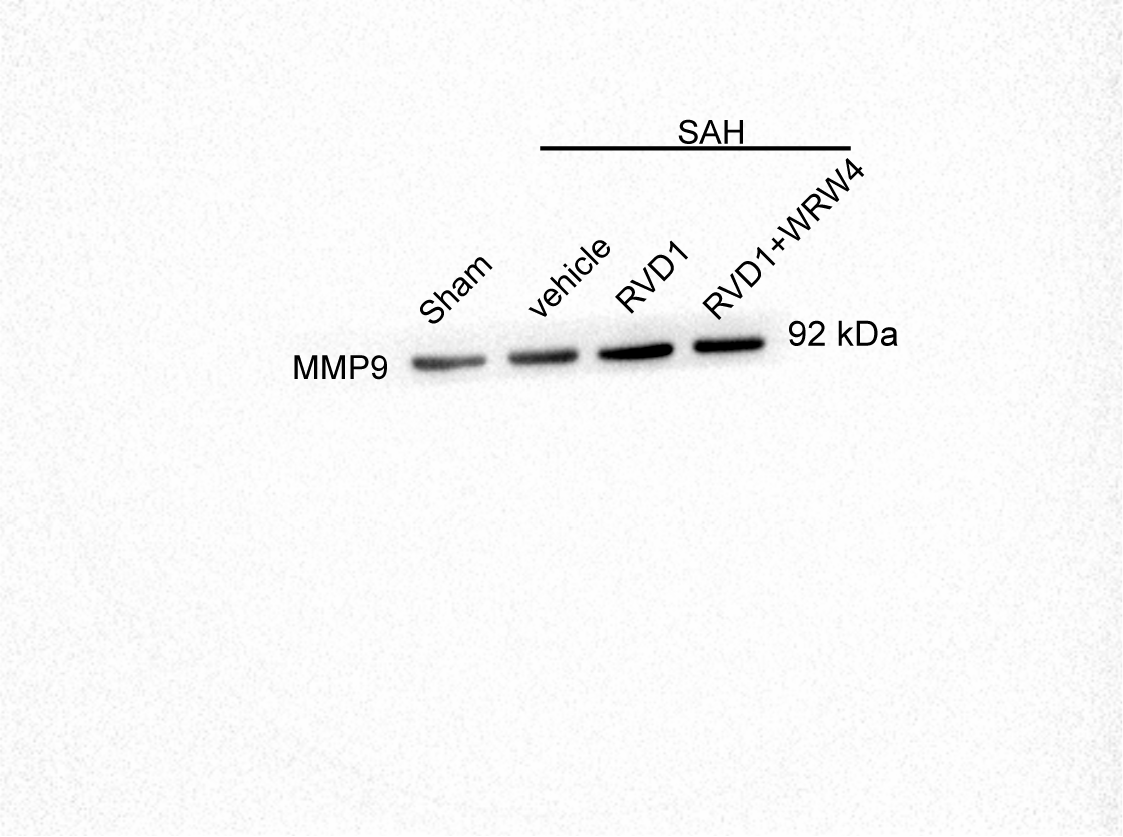

Supplement: Supplementary file 1 [file datasheet1.zip › A20-original images/A20/A20-4-Administrator 2020-06-17 07 ╩▒ 37 ╖╓_Exposure_17.0sec.tif]

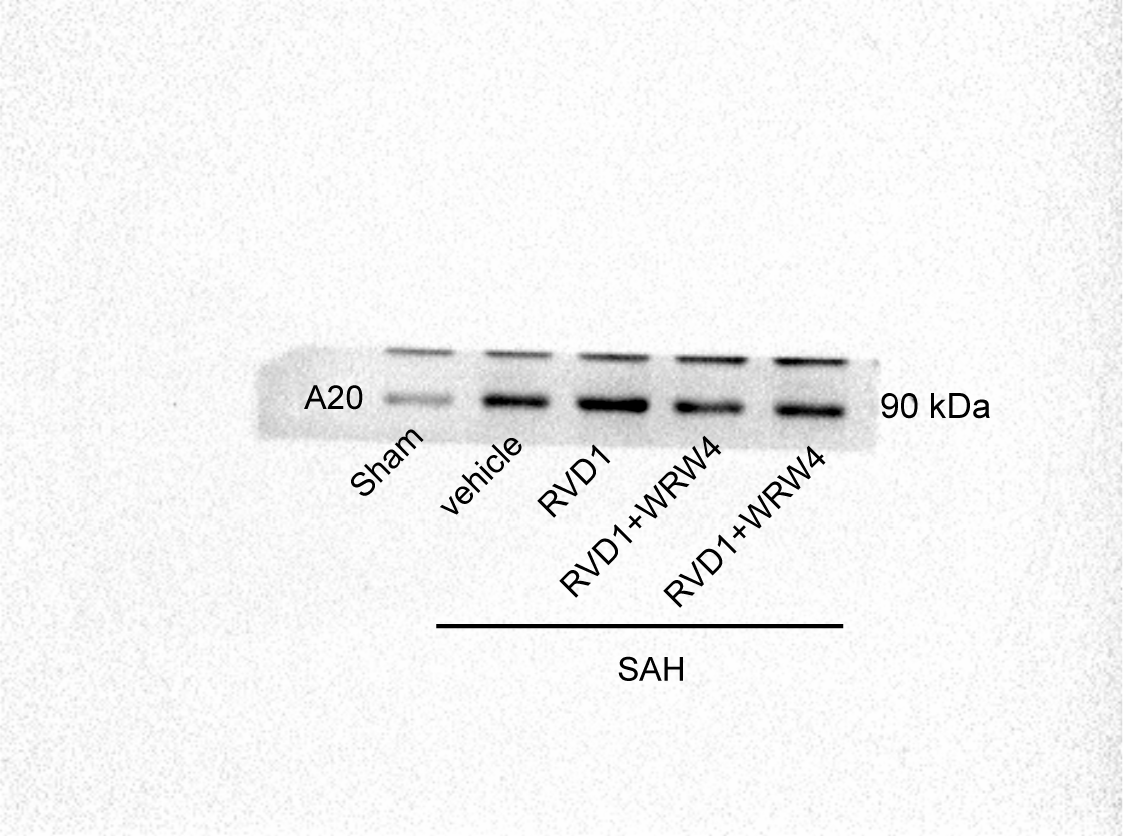

Supplement: Supplementary file 1 [file datasheet1.zip › A20-original images/A20/A20-5-Administrator 2020-07-04 23 ╩▒ 50 ╖╓-.tif]

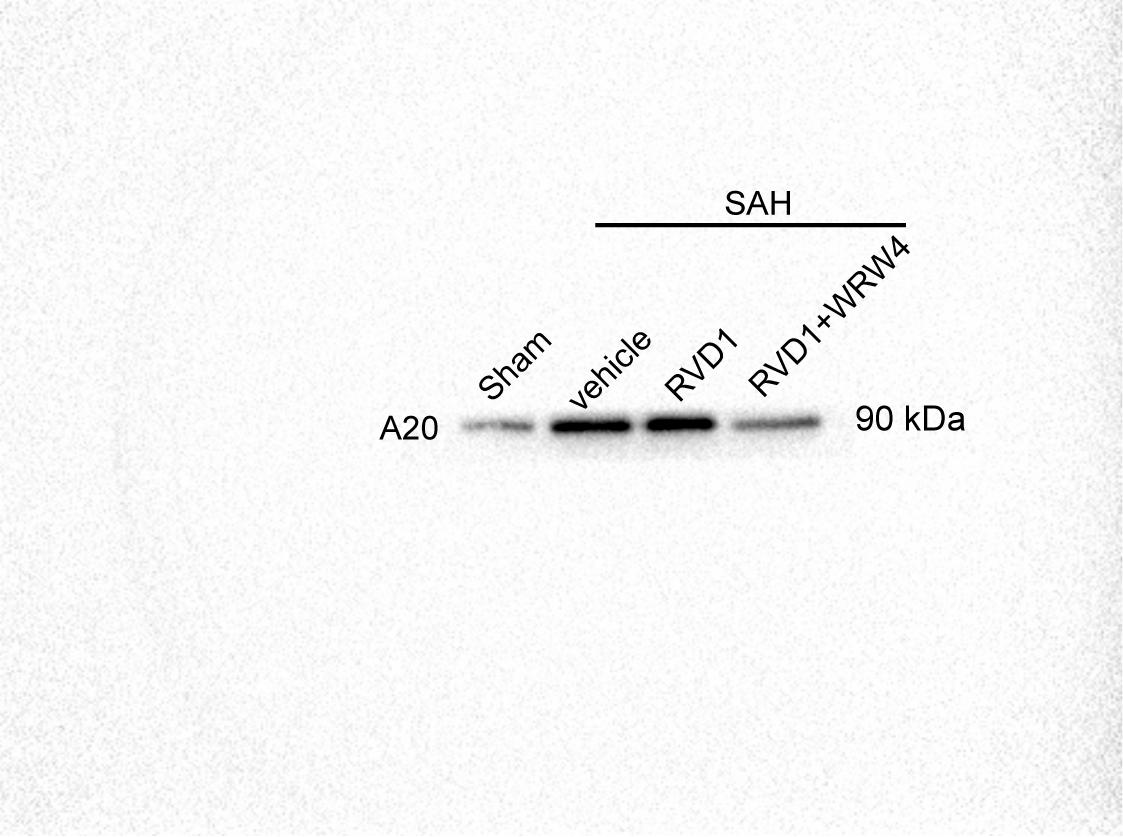

Supplement: Supplementary file 1 [file datasheet1.zip › A20-original images/A20/A20-6-Administrator 2020-06-17 07 ╩▒ 22 ╖╓_Exposure_36.0sec.tif]

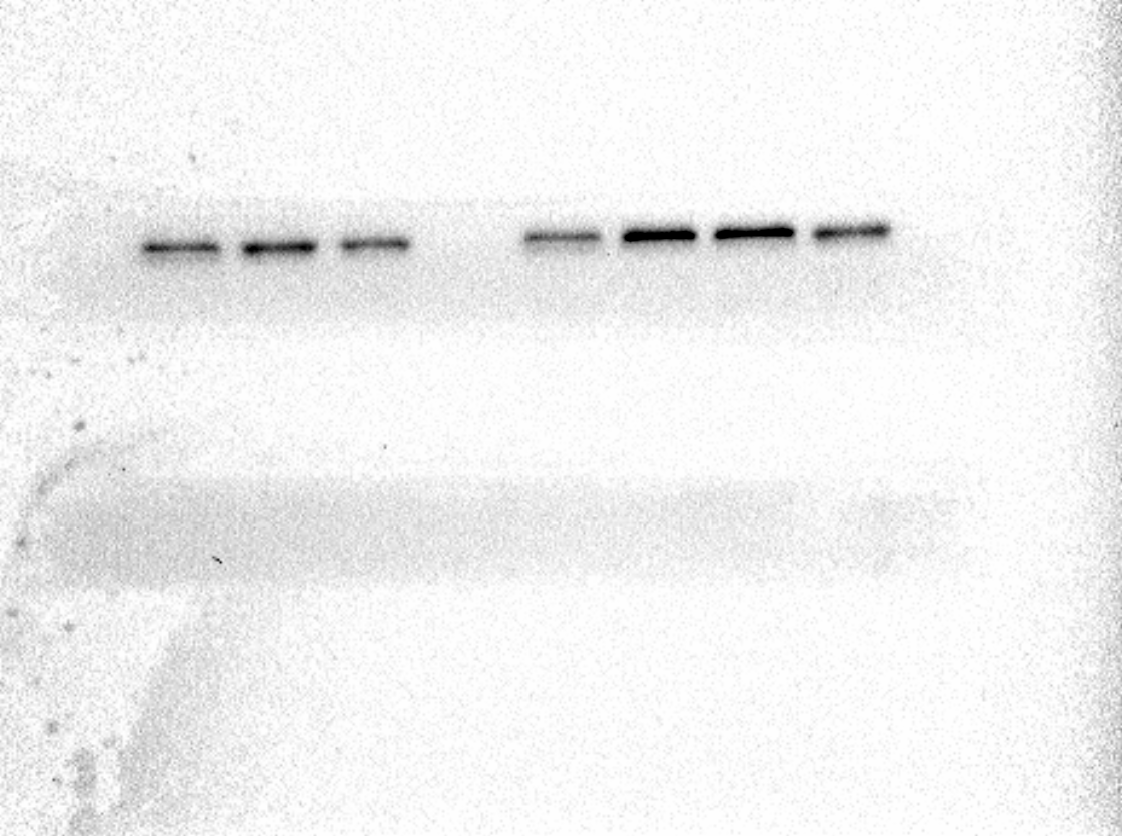

Supplement: Supplementary file 1 [file datasheet1.zip › A20-original images/A20-1Administrator 2019-12-19 09 ╩▒ 54 ╖╓_Exposure_198.0sec.tif]

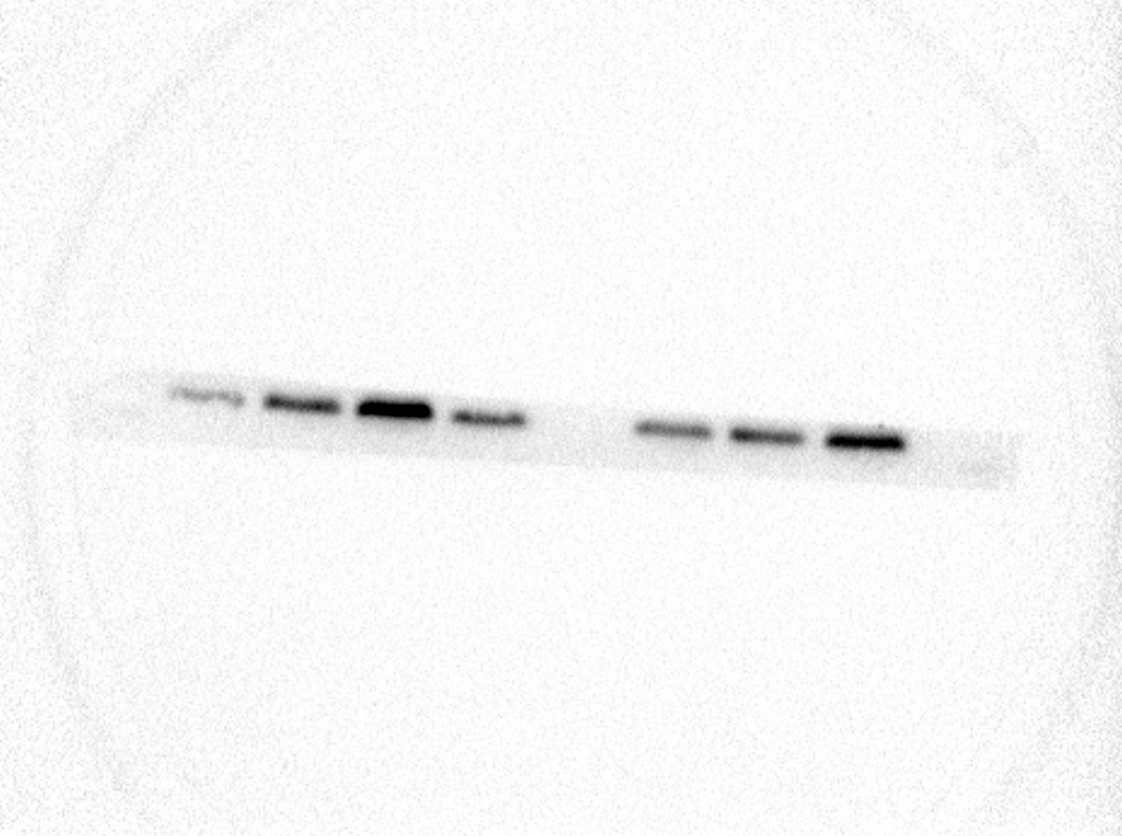

Supplement: Supplementary file 1 [file datasheet1.zip › A20-original images/A20-2Administrator 2020-06-16 23 ╩▒ 46 ╖╓_Exposure_53.5sec.tif]

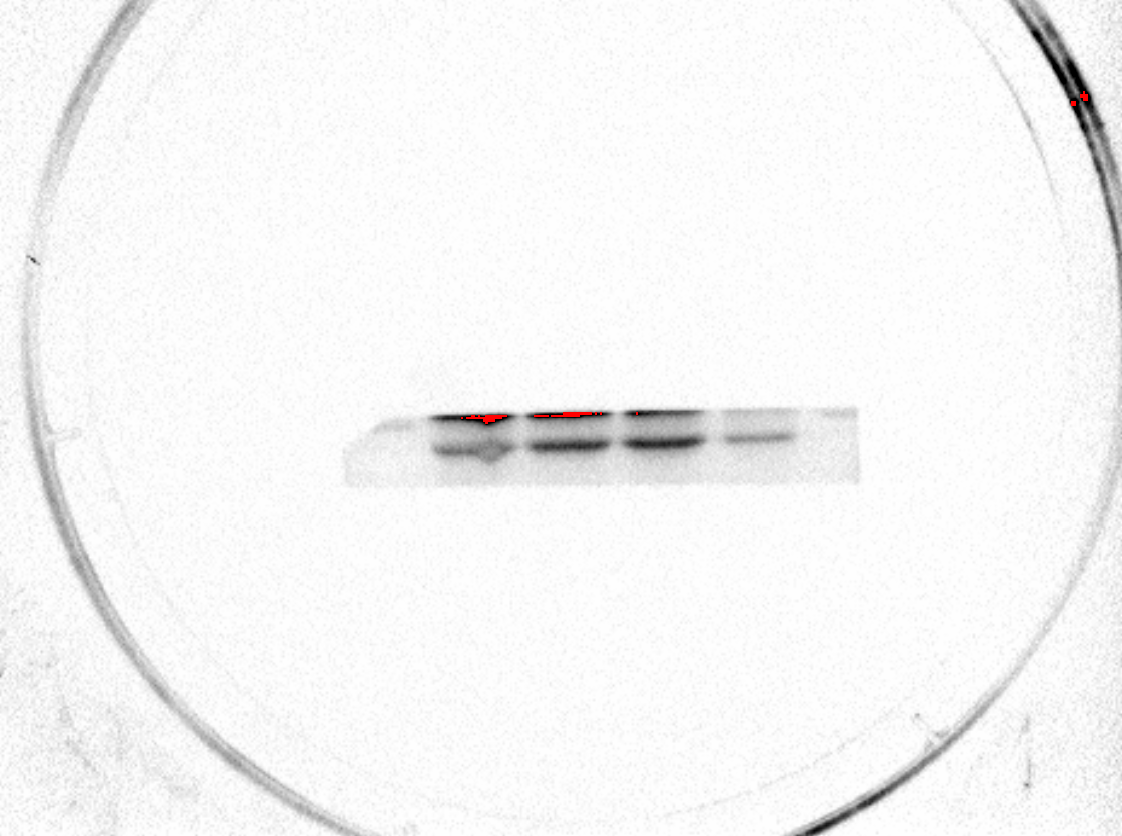

Supplement: Supplementary file 1 [file datasheet1.zip › A20-original images/A20-3Administrator 2020-06-16 23 ╩▒ 59 ╖╓_Exposure_105.5sec.tif]

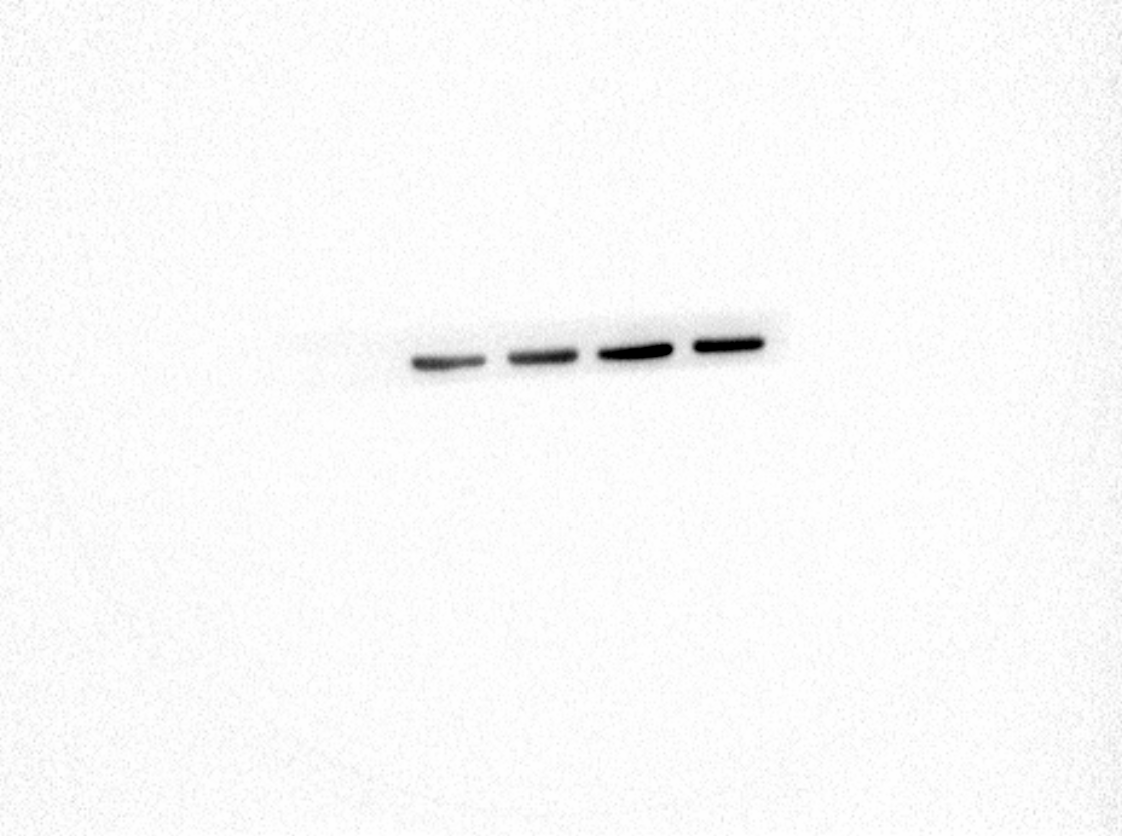

Supplement: Supplementary file 1 [file datasheet1.zip › A20-original images/A20-4-Administrator 2020-06-17 07 ╩▒ 37 ╖╓_Exposure_17.0sec.tif]

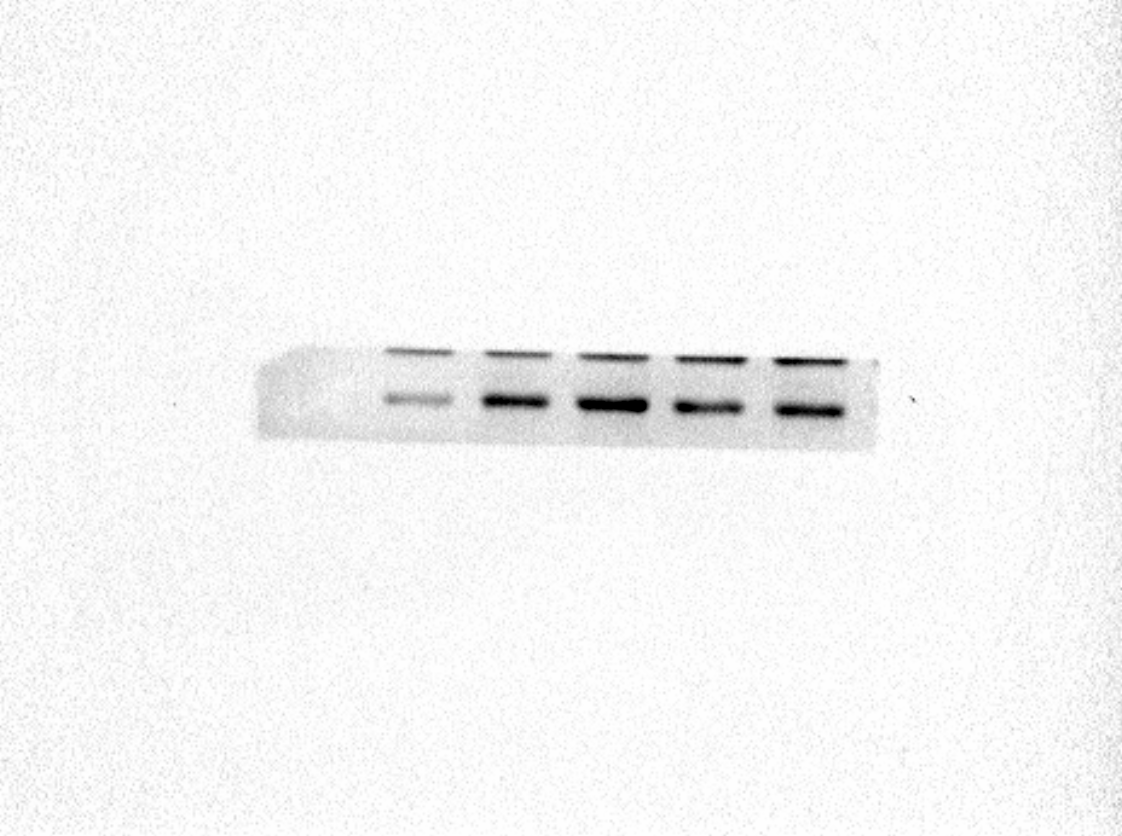

Supplement: Supplementary file 1 [file datasheet1.zip › A20-original images/A20-5-Administrator 2020-07-04 23 ╩▒ 50 ╖╓-.tif]

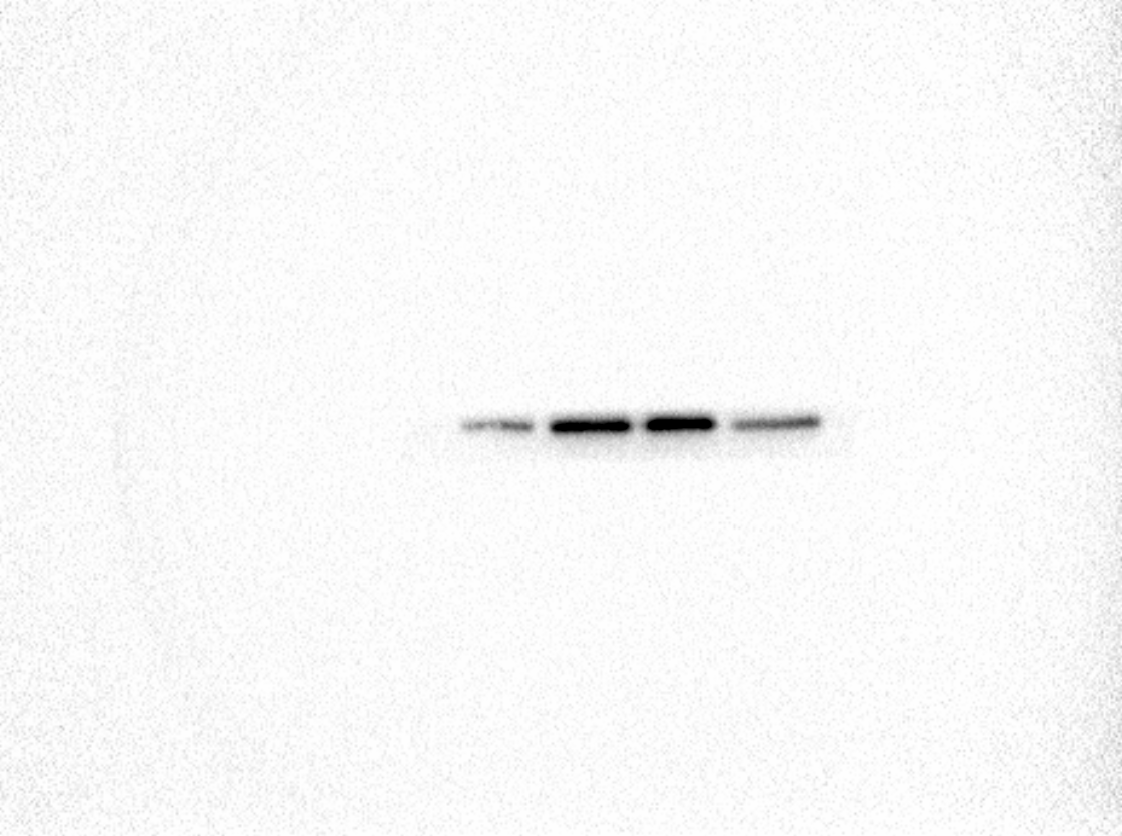

Supplement: Supplementary file 1 [file datasheet1.zip › A20-original images/A20-6-Administrator 2020-06-17 07 ╩▒ 22 ╖╓_Exposure_36.0sec.tif]

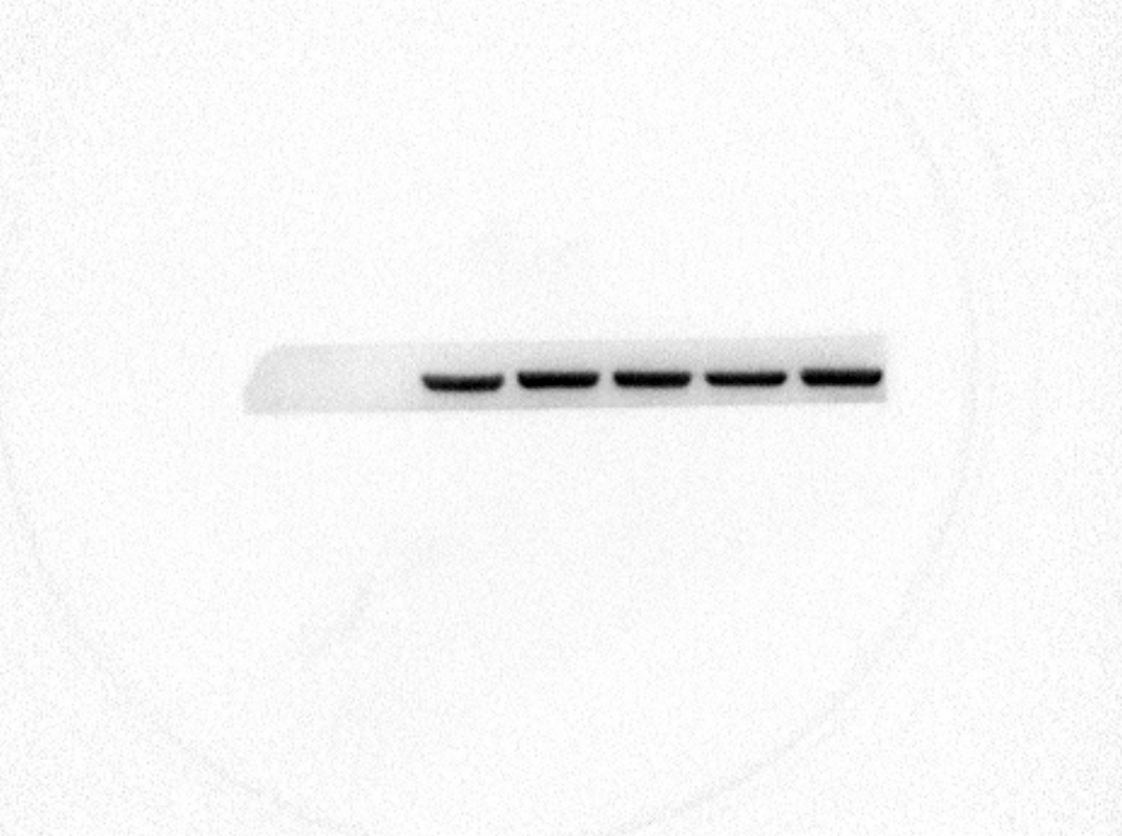

Supplement: Supplementary file 1 [file datasheet1.zip › A20-original images/Administrator 2020-07-03 23 ╩▒ 36 ╖╓_Exposure_4.8sec.tif]

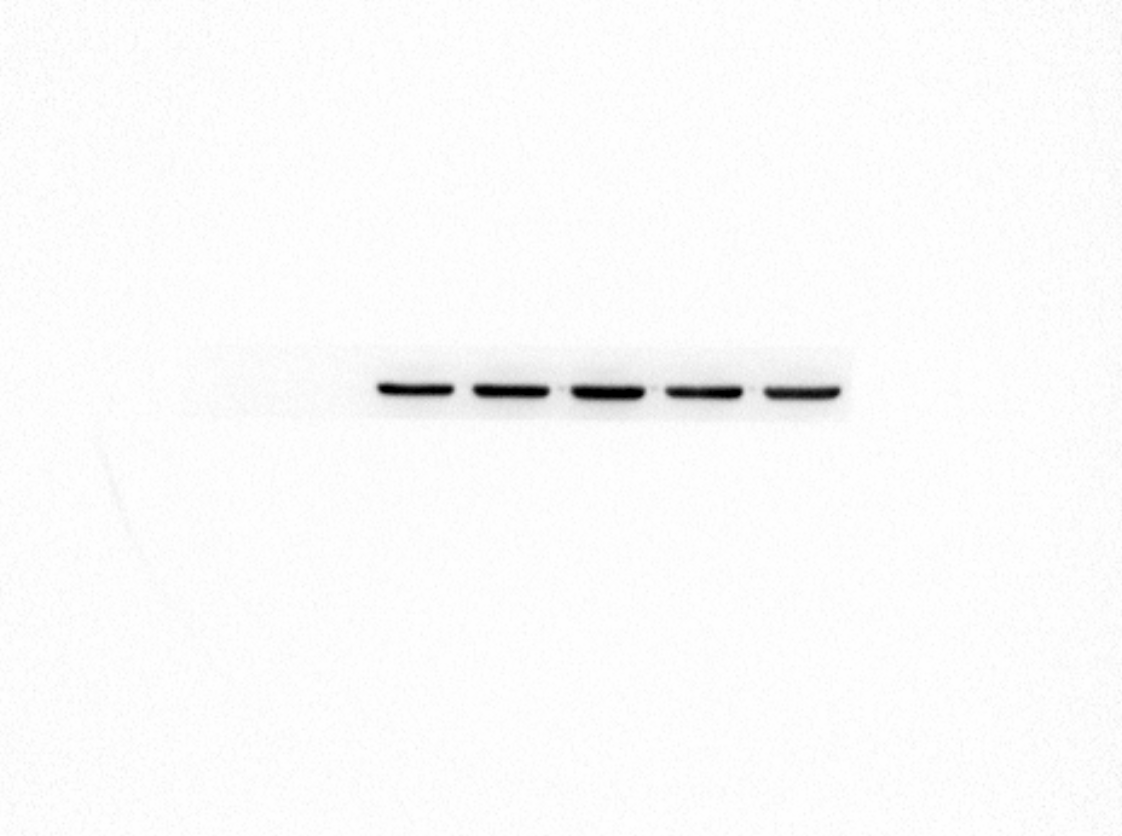

Supplement: Supplementary file 1 [file datasheet1.zip › A20-original images/Administrator 2020-07-04 01 ╩▒ 47 ╖╓_Exposure_5.0sec.tif]

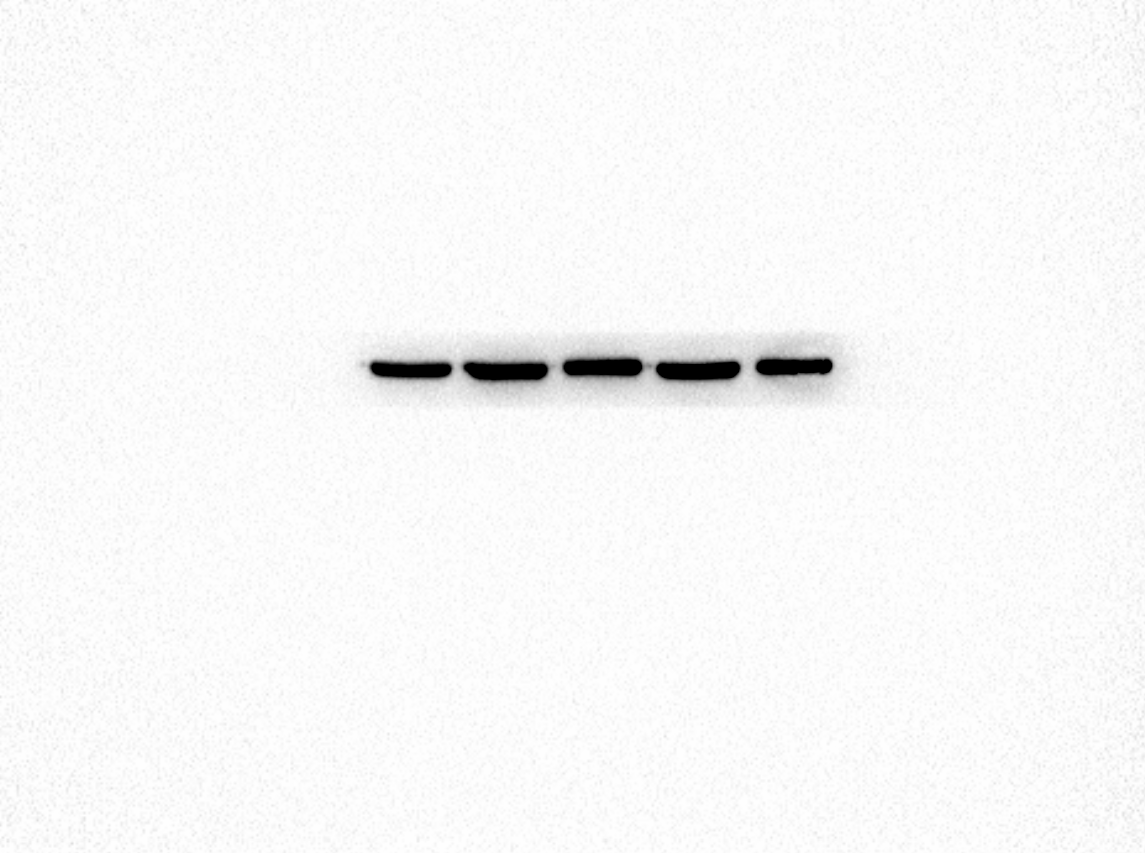

Supplement: Supplementary file 1 [file datasheet1.zip › A20-original images/Administrator 2020-07-04 03 ╩▒ 07 ╖╓_Exposure_2.3sec.tif]

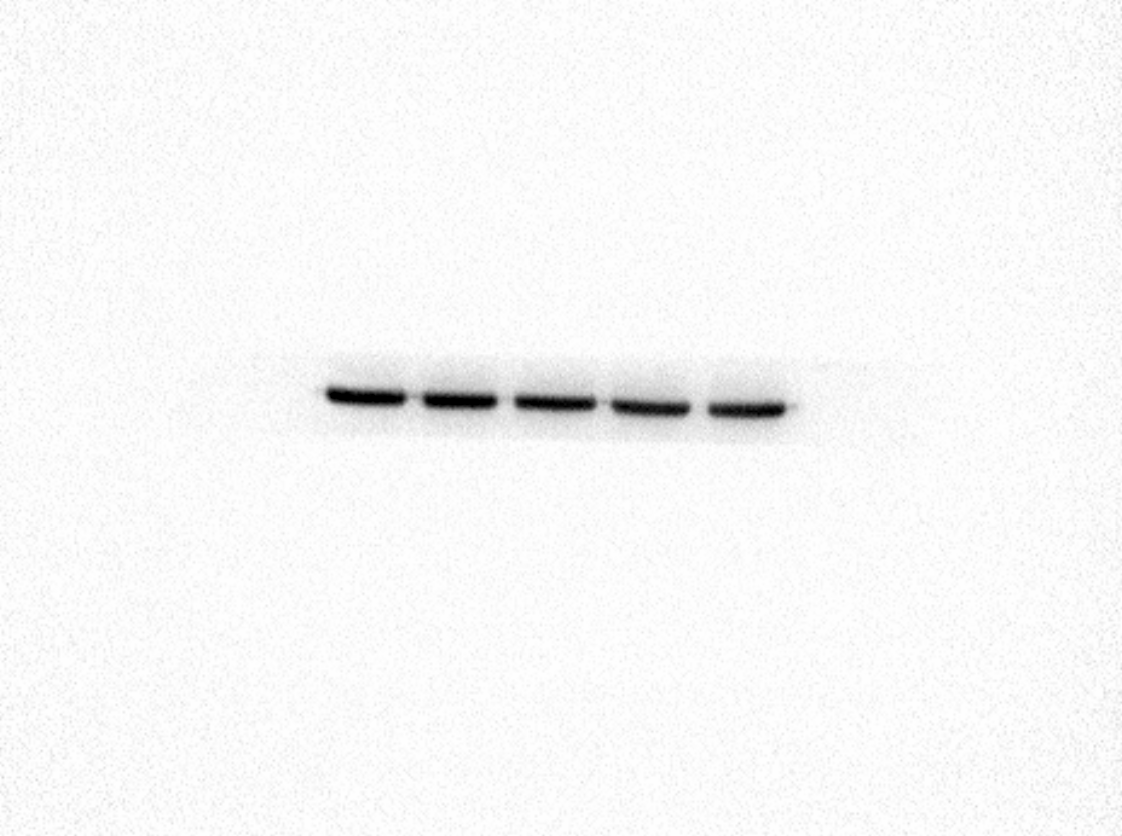

Supplement: Supplementary file 1 [file datasheet1.zip › A20-original images/Administrator 2020-07-09 22 ╩▒ 48 ╖╓_Exposure_4.8sec--actin.tif]

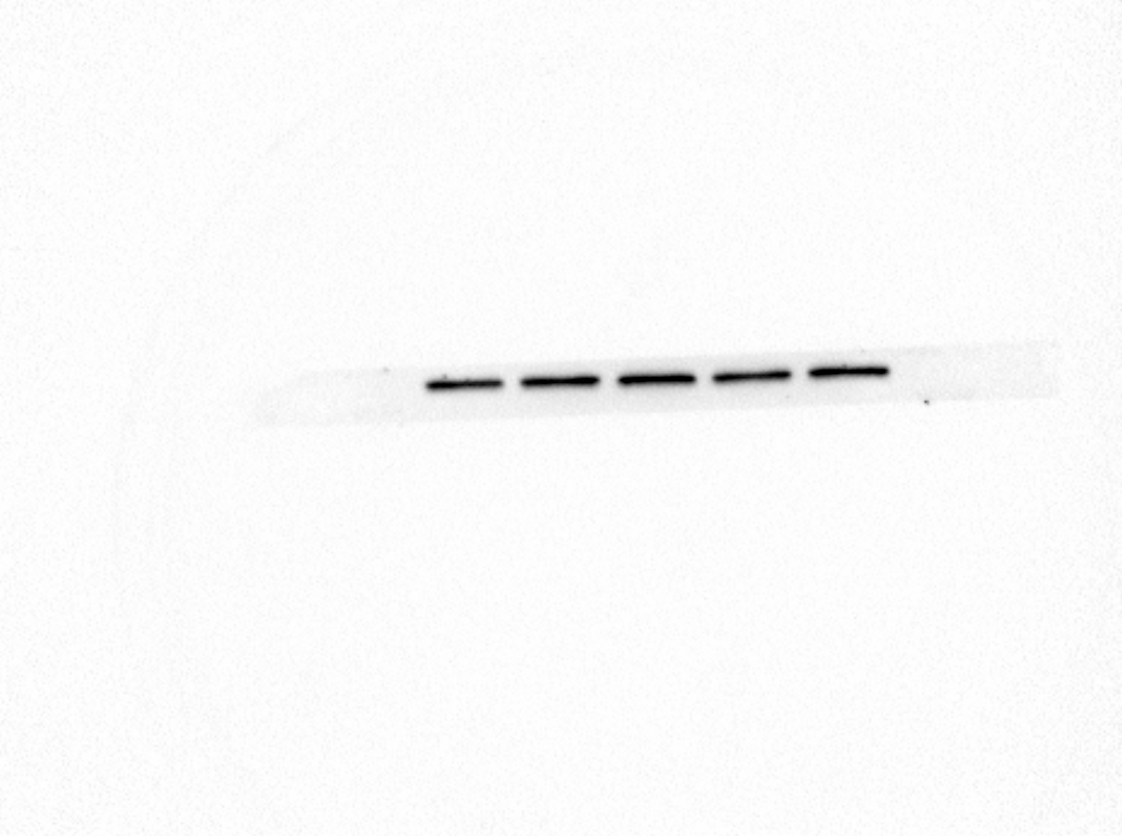

Supplement: Supplementary file 1 [file datasheet1.zip › A20-original images/Administrator 2020-07-10 00 ╩▒ 22 ╖╓_Exposure_27.2sec-actin.tif]

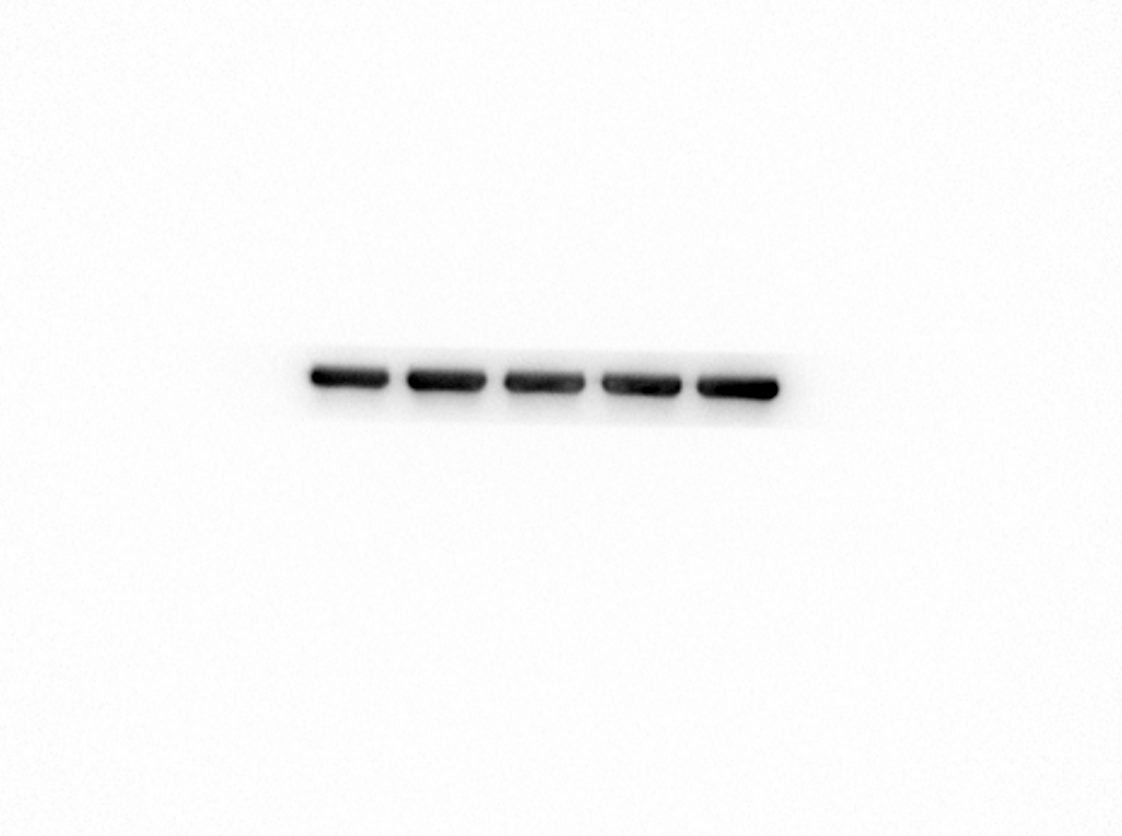

Supplement: Supplementary file 1 [file datasheet1.zip › A20-original images/Administrator 2020-07-10 22 ╩▒ 45 ╖╓_Exposure_1.0sec.tif]

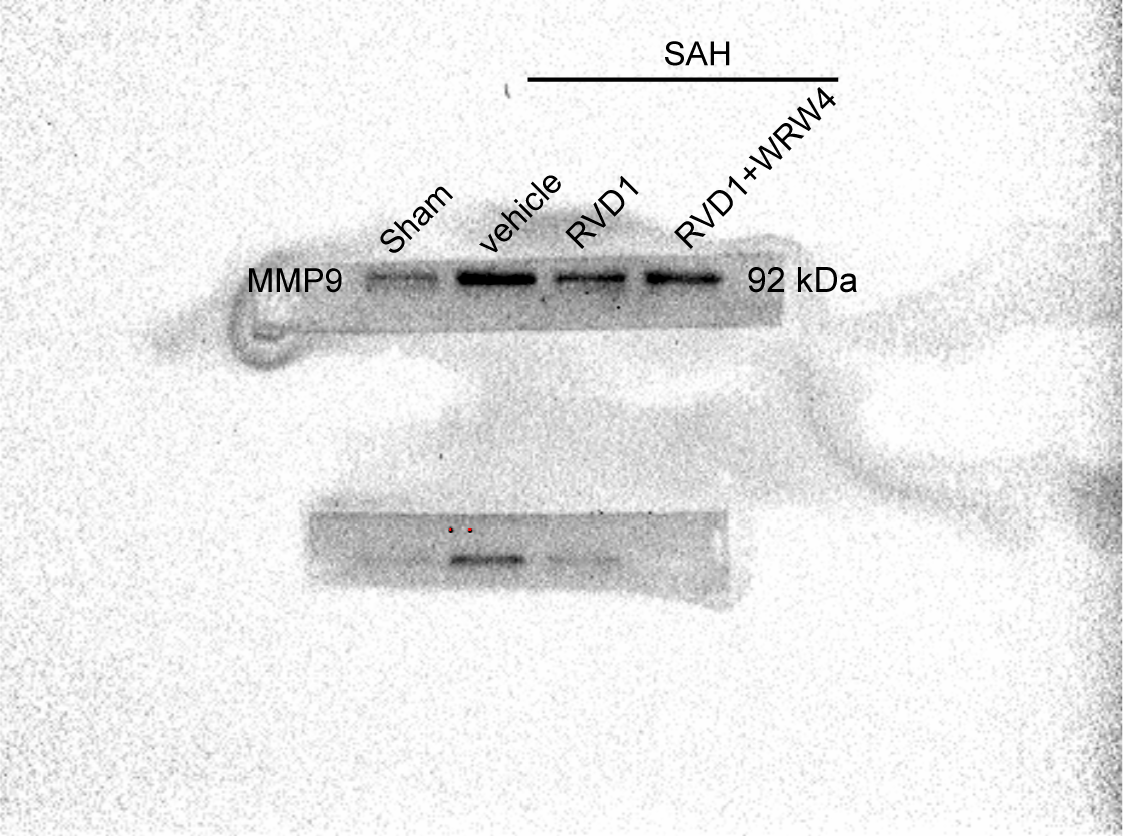

Supplement: Supplementary file 2 [file datasheet2.zip › MMP9-original images/MMP9-1/MMP9-1-Administrator 2020-01-09 06 ╩▒ 51 ╖╓_Exposure_400.0sec.tif]

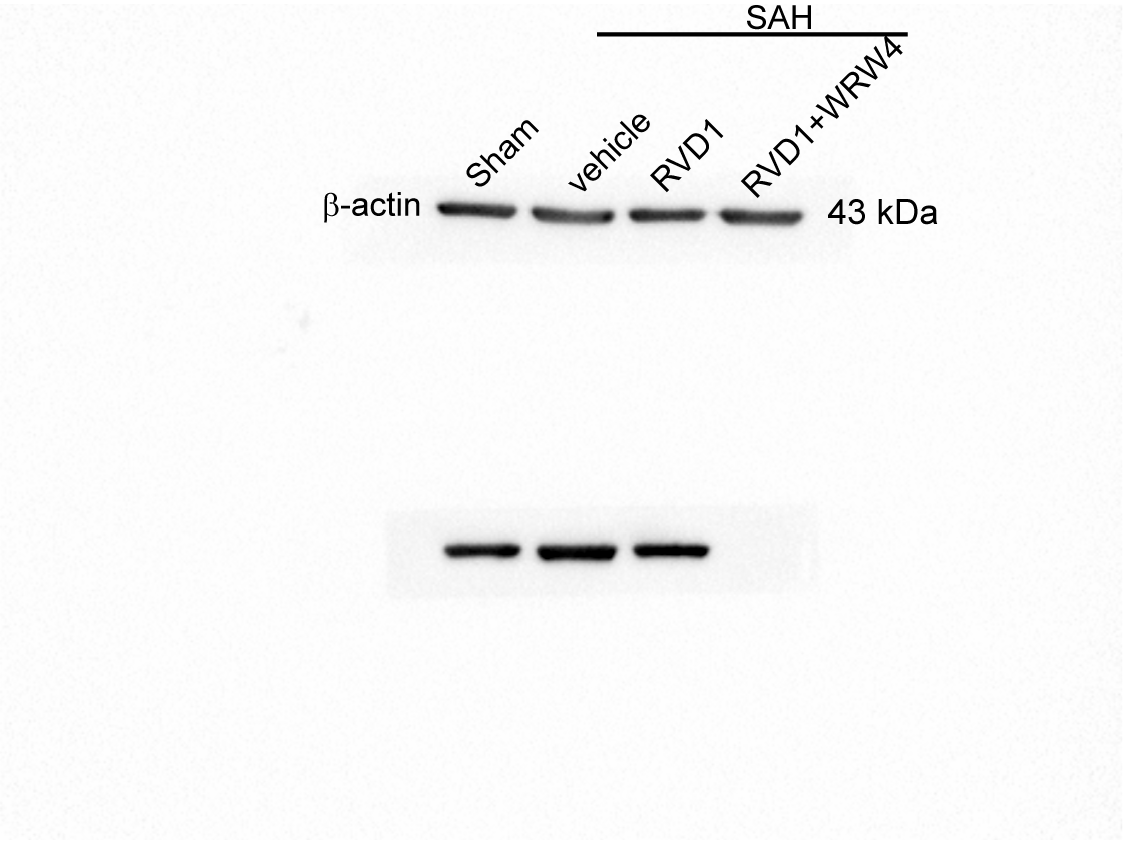

Supplement: Supplementary file 2 [file datasheet2.zip › MMP9-original images/MMP9-1/b-actin-1-Administrator 2020-01-09 06 ╩▒ 59 ╖╓_Exposure_5.0sec.tif]

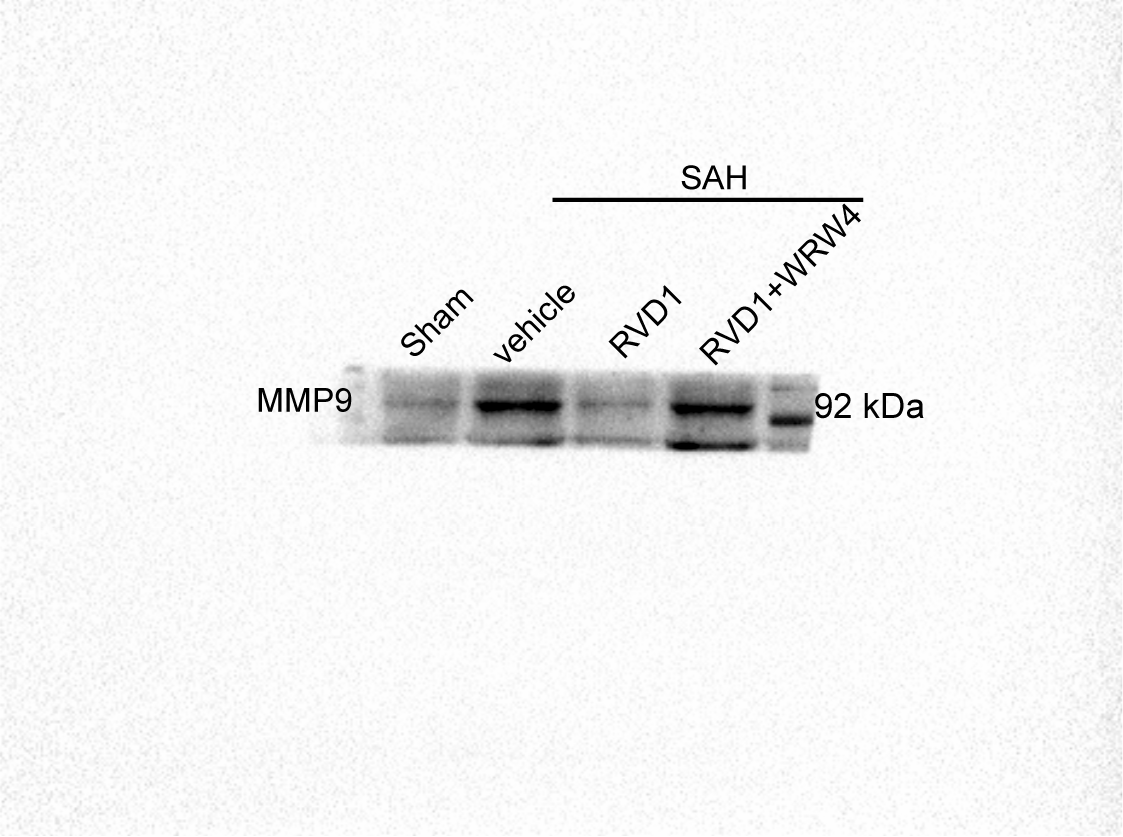

Supplement: Supplementary file 2 [file datasheet2.zip › MMP9-original images/MMP9-2/MMP9-Administrator 2020-06-14 08 ╩▒ 43 ╖╓_Exposure_41.2sec.tif]

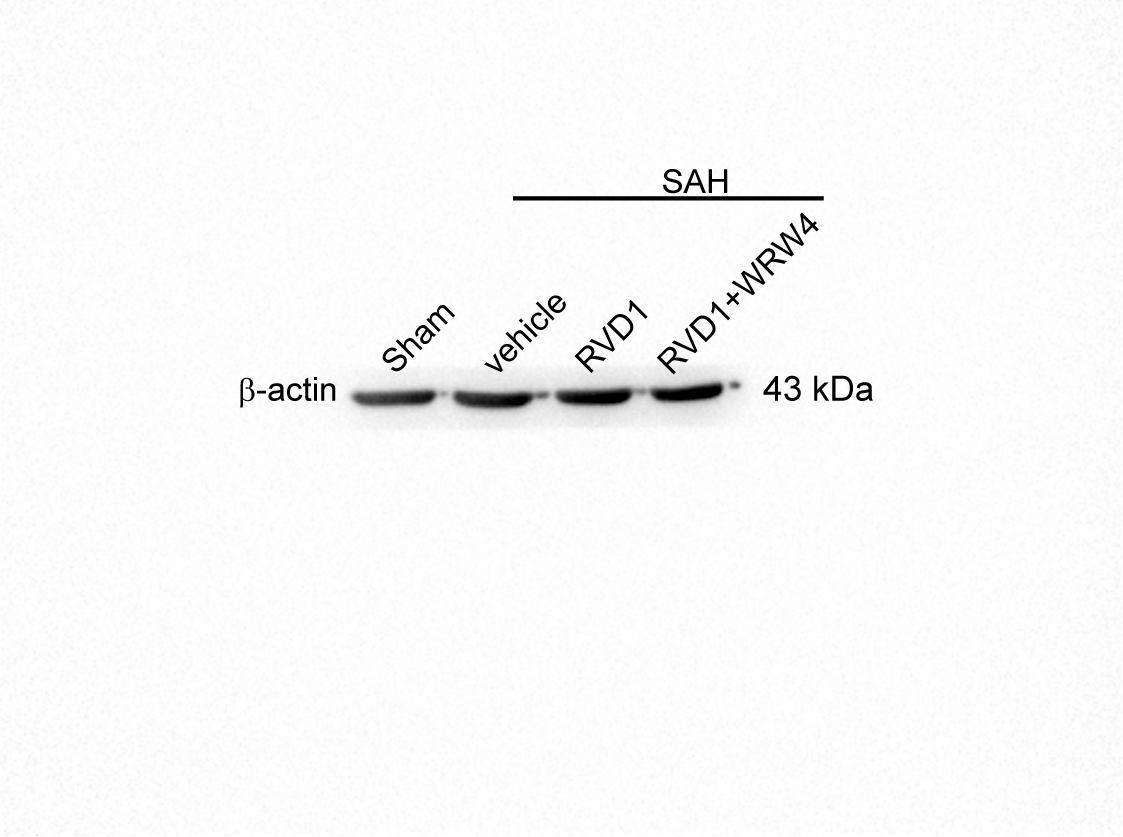

Supplement: Supplementary file 2 [file datasheet2.zip › MMP9-original images/MMP9-2/b-actin-Administrator 2020-06-14 08 ╩▒ 48 ╖╓_Exposure_4.0sec.tif]

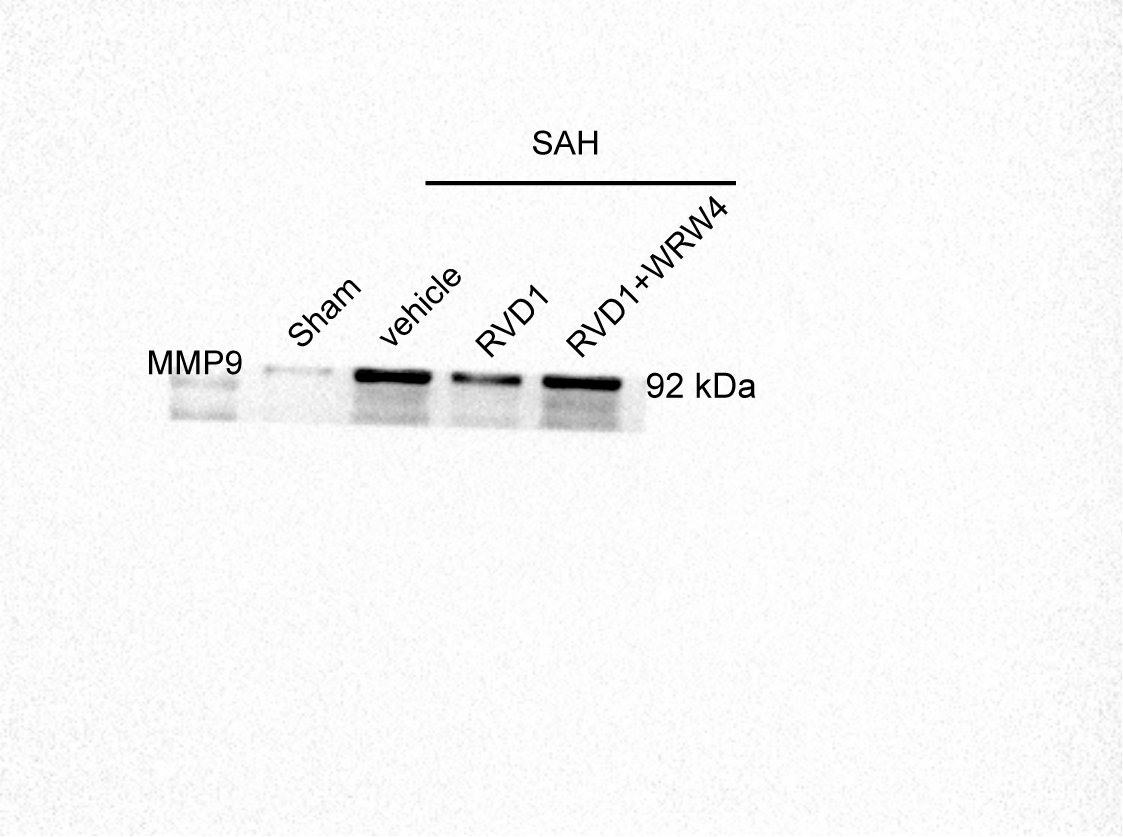

Supplement: Supplementary file 2 [file datasheet2.zip › MMP9-original images/MMP9-3/MMP9-Administrator 2020-06-14 23 ╩▒ 20 ╖╓_Exposure_17.2sec.tif]

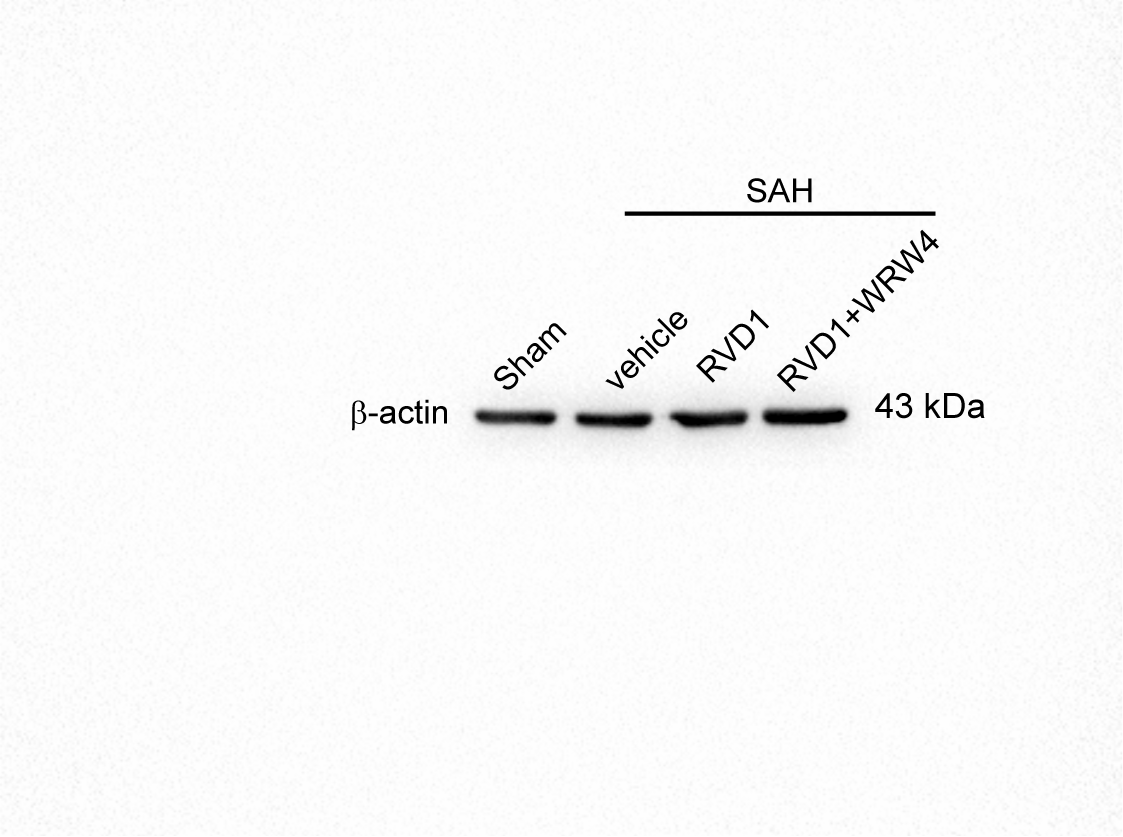

Supplement: Supplementary file 2 [file datasheet2.zip › MMP9-original images/MMP9-3/b-actin -Administrator 2020-06-15 00 ╩▒ 27 ╖╓-1_Exposure_3.8sec--.tif]

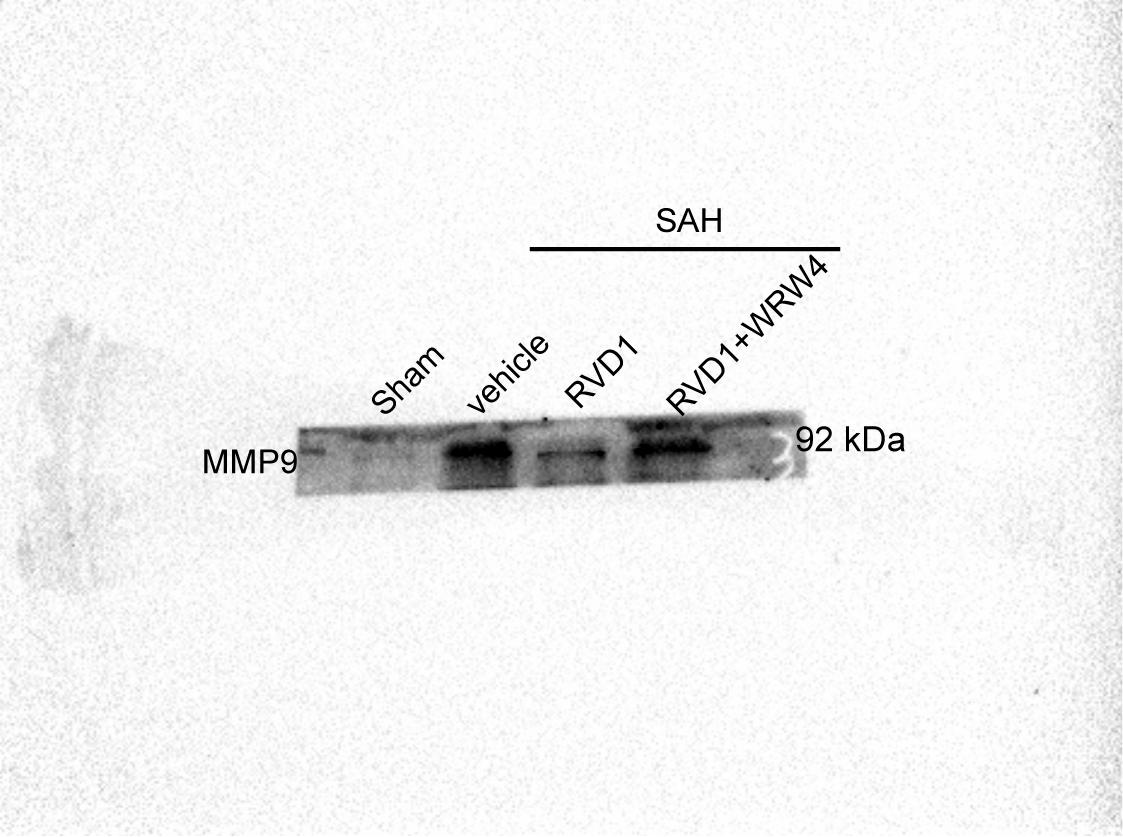

Supplement: Supplementary file 2 [file datasheet2.zip › MMP9-original images/MMP9-4/MMP9-Administrator 2020-06-15 09 ╩▒ 32 ╖╓_Exposure_200.0sec.tif]

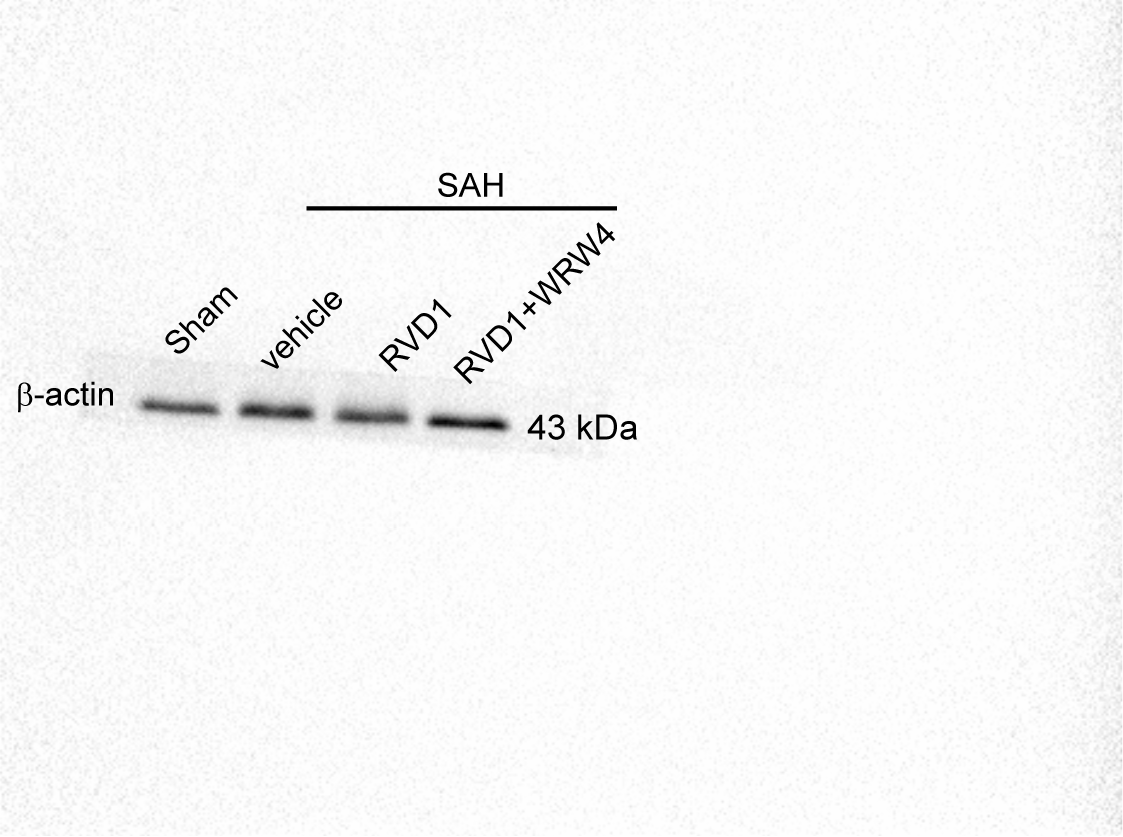

Supplement: Supplementary file 2 [file datasheet2.zip › MMP9-original images/MMP9-4/b-actin-Administrator 2020-06-15 00 ╩▒ 09 ╖╓_Exposure_83.4sec.tif]

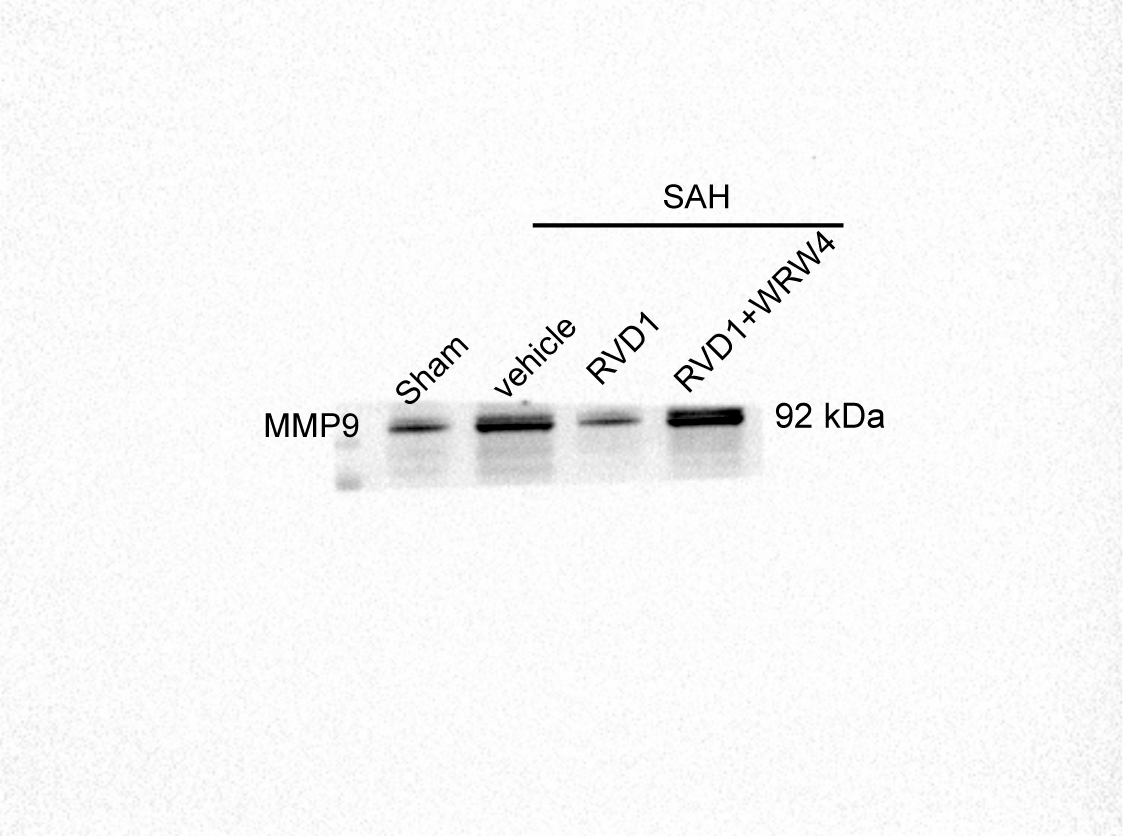

Supplement: Supplementary file 2 [file datasheet2.zip › MMP9-original images/MMP9-5/MMP9-5-Administrator 2020-06-16 00 ╩▒ 01 ╖╓_Exposure_12.0sec.tif]

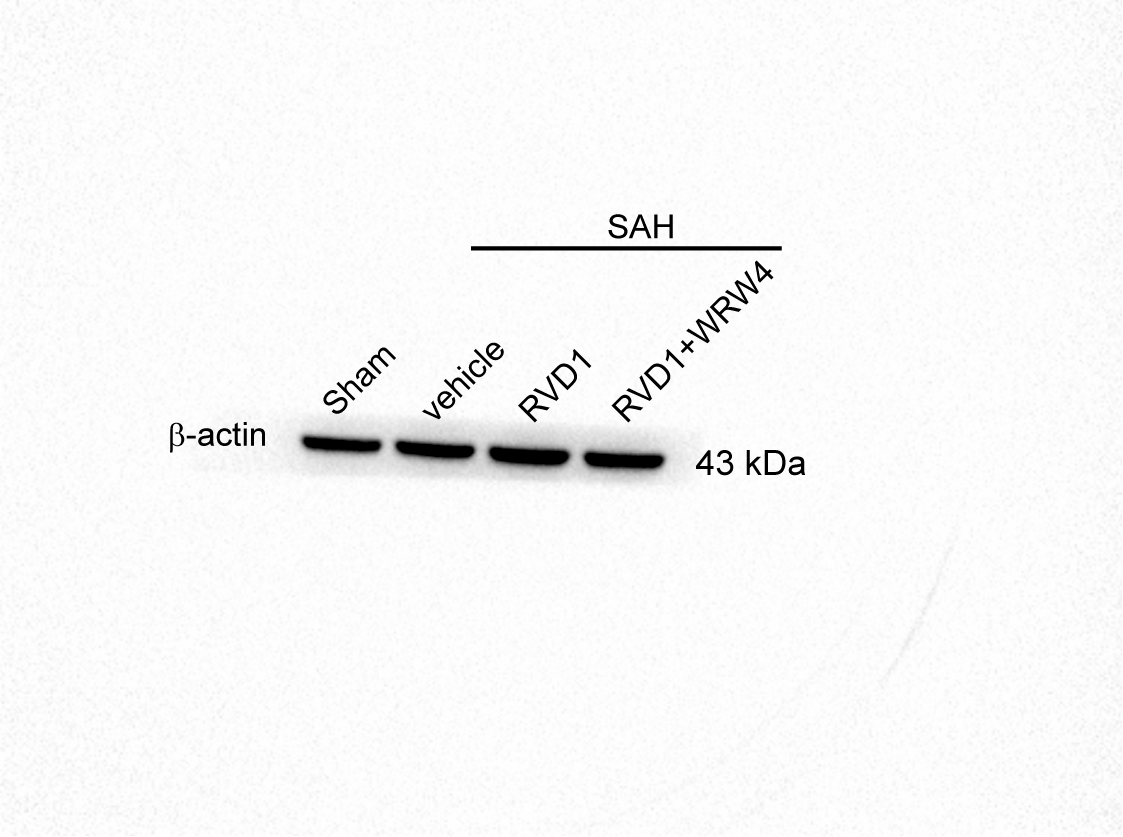

Supplement: Supplementary file 2 [file datasheet2.zip › MMP9-original images/MMP9-5/b-actin-Administrator 2020-06-16 23 ╩▒ 17 ╖╓_Exposure_11.1sec.tif]

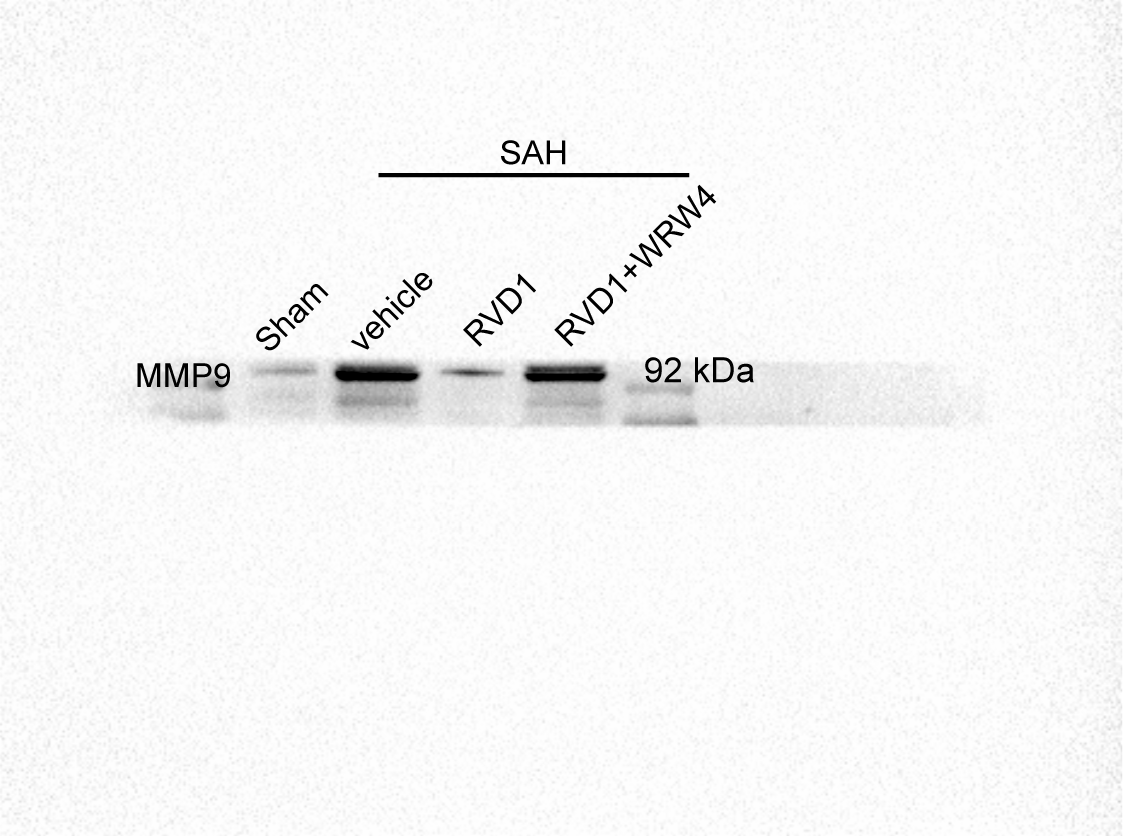

Supplement: Supplementary file 2 [file datasheet2.zip › MMP9-original images/MMP9-6/MMP9-6-Administrator 2020-06-16 00 ╩▒ 34 ╖╓_Exposure_12.0sec.tif]

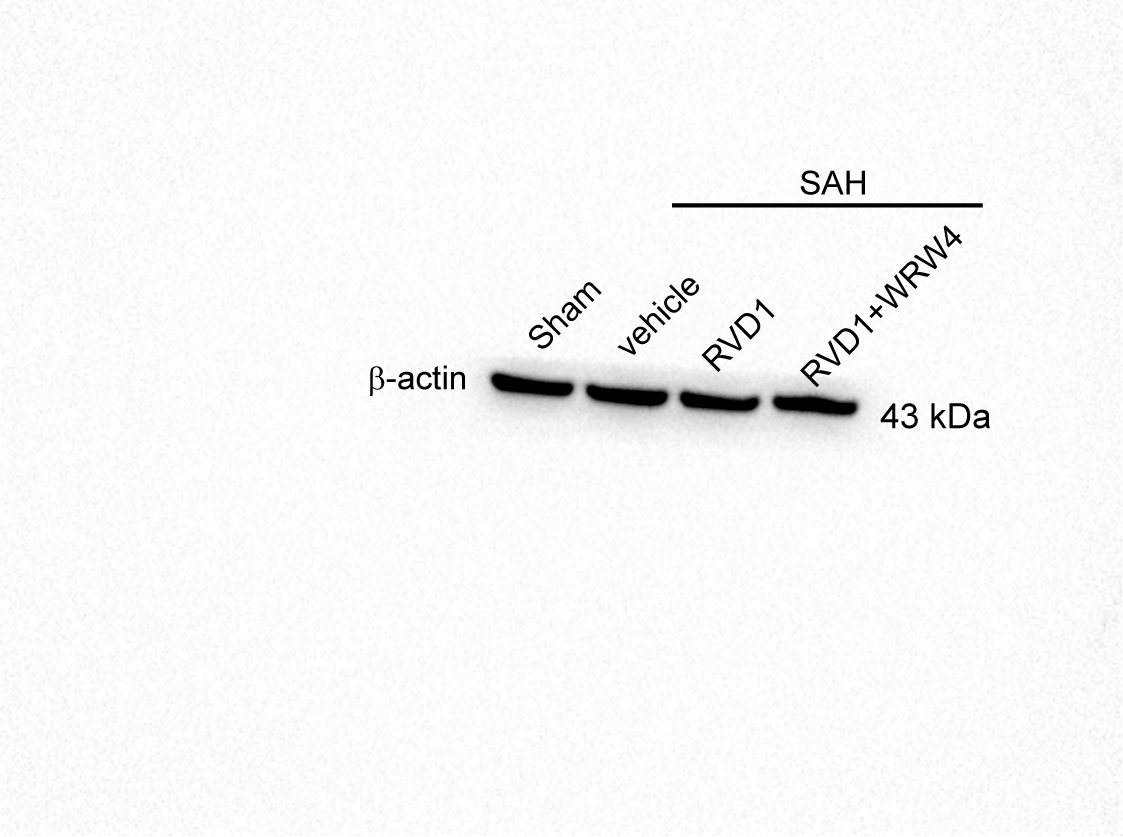

Supplement: Supplementary file 2 [file datasheet2.zip › MMP9-original images/MMP9-6/b-actin-Administrator 2020-06-16 23 ╩▒ 35 ╖╓_Exposure_7.1sec.tif]

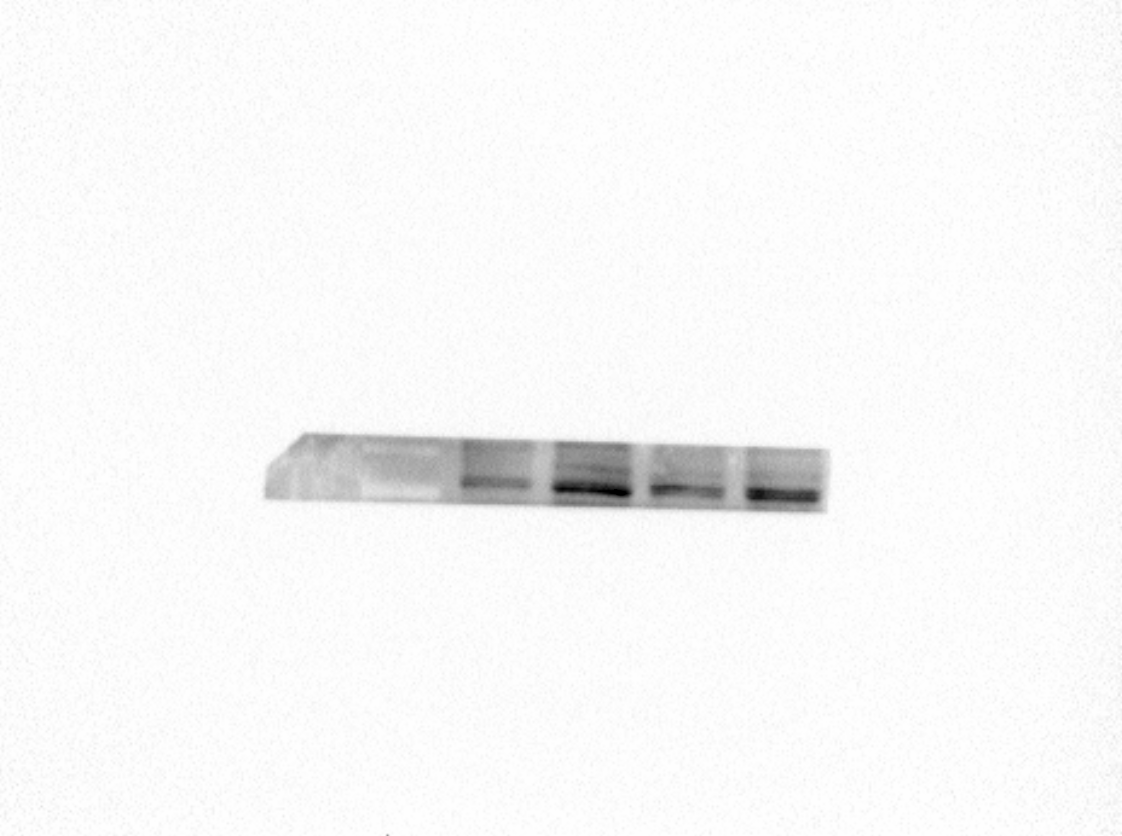

Supplement: Supplementary file 3 [file datasheet3.zip › NLRP3-original images/NLRP3-1-Administrator 2020-06-13 09 ╩▒ 19 ╖╓_Exposure_31.1sec.tif]

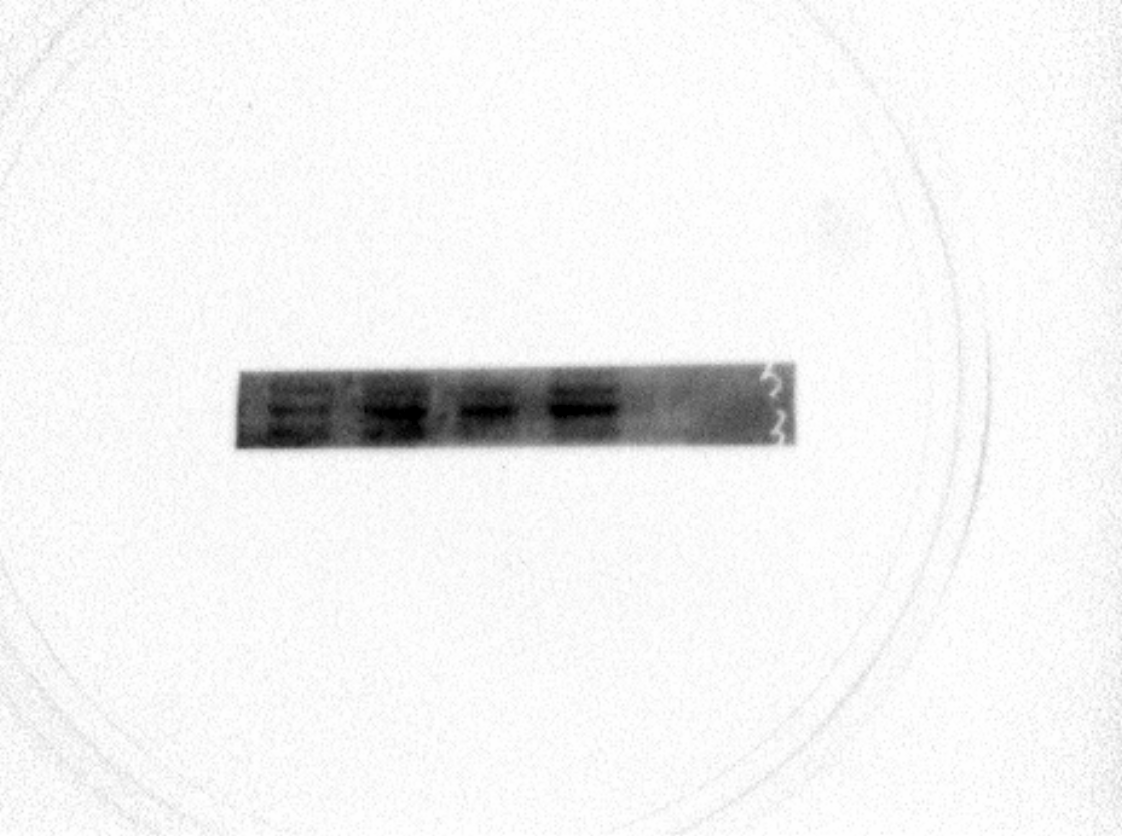

Supplement: Supplementary file 3 [file datasheet3.zip › NLRP3-original images/NLRP3-2-Administrator 2020-06-17 08 ╩▒ 17 ╖╓_Exposure_67.7sec.tif]

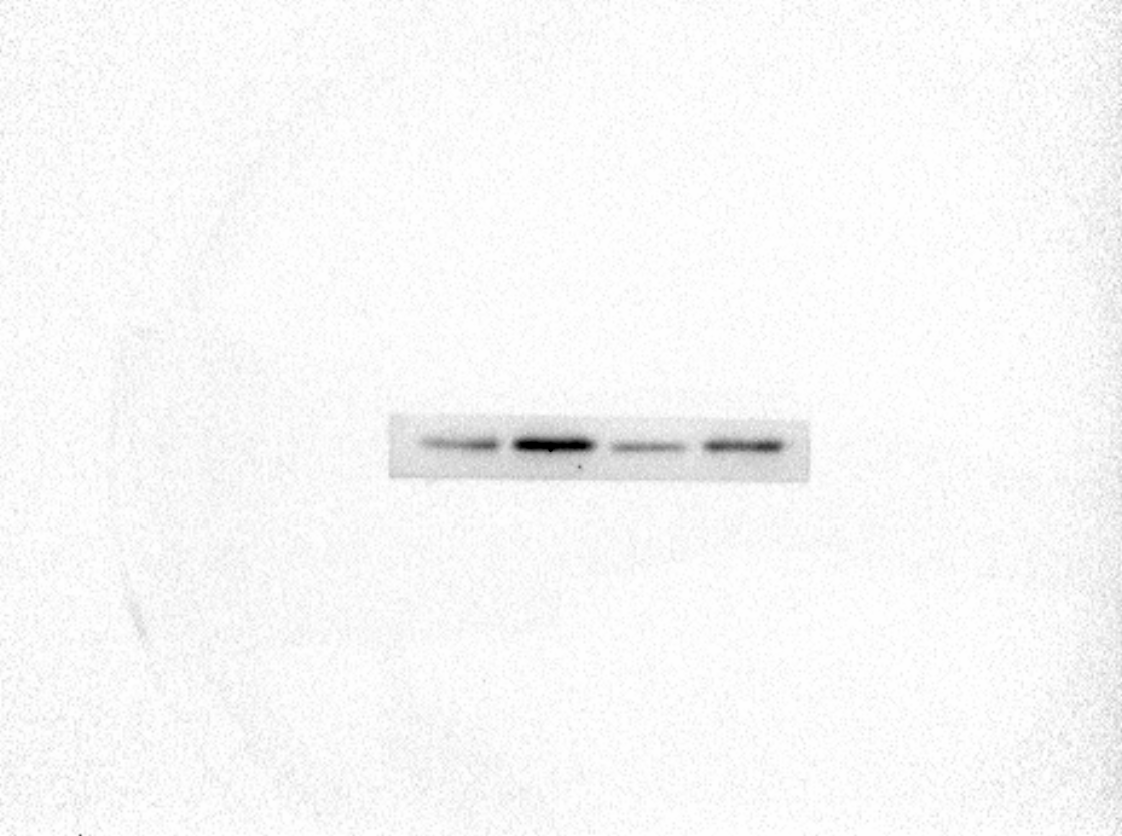

Supplement: Supplementary file 3 [file datasheet3.zip › NLRP3-original images/NLRP3-3-Administrator 2020-06-17 23 ╩▒ 05 ╖╓_Exposure_133.9sec.tif]

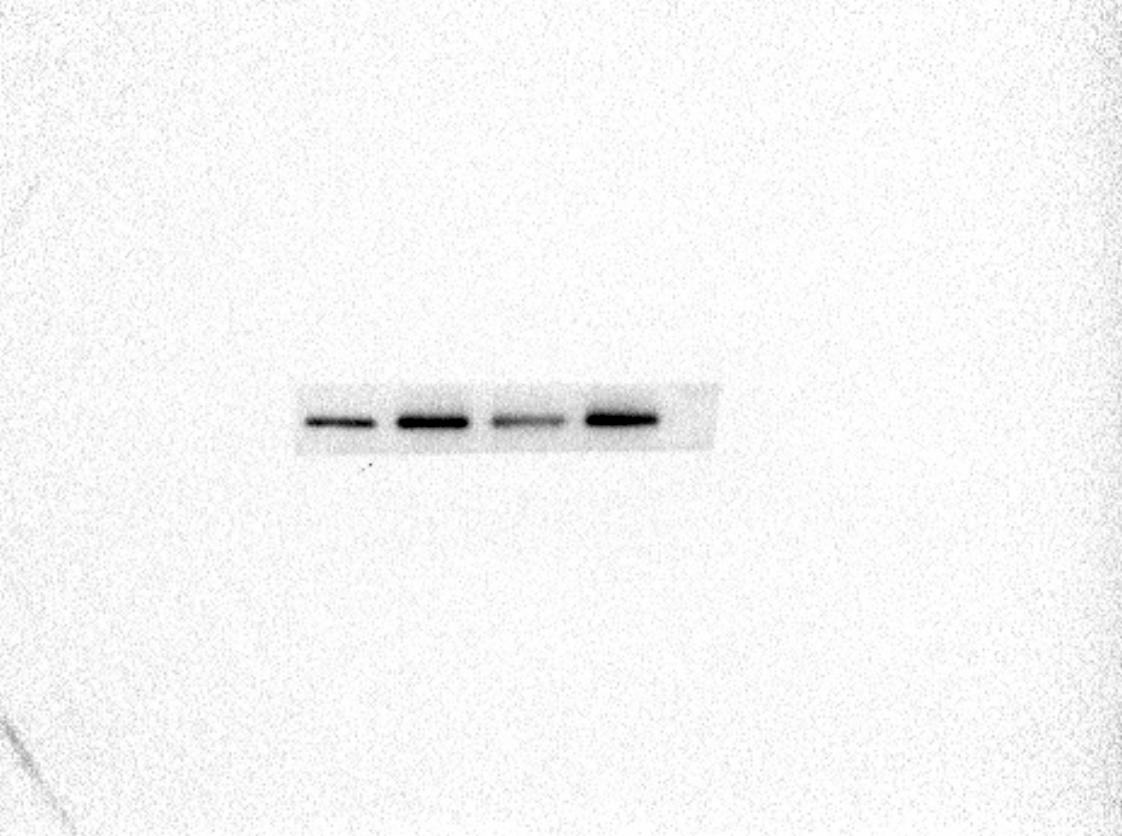

Supplement: Supplementary file 3 [file datasheet3.zip › NLRP3-original images/NLRP3-4-Administrator 2020-06-18 23 ╩▒ 15 ╖╓_Exposure_59.3sec.tif]

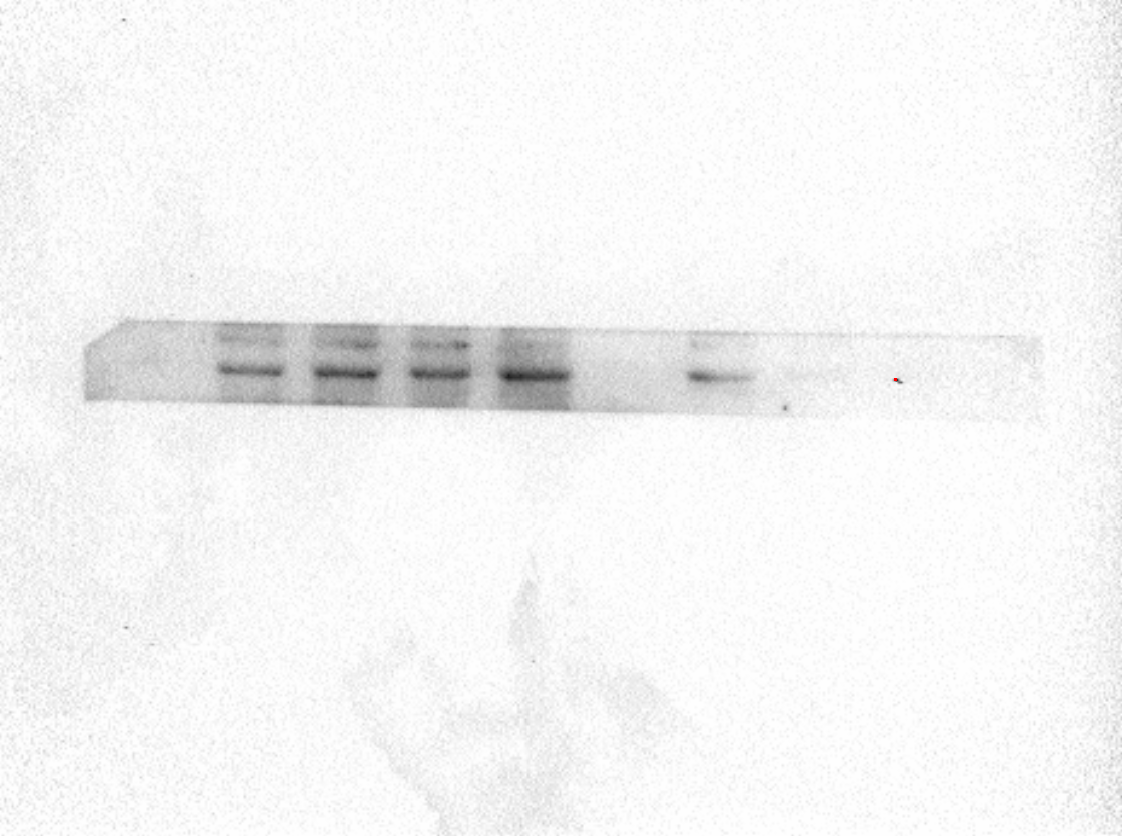

Supplement: Supplementary file 3 [file datasheet3.zip › NLRP3-original images/NLRP3-5-Administrator 2020-06-19 00 ╩▒ 30 ╖╓_Exposure_222.6sec.tif]

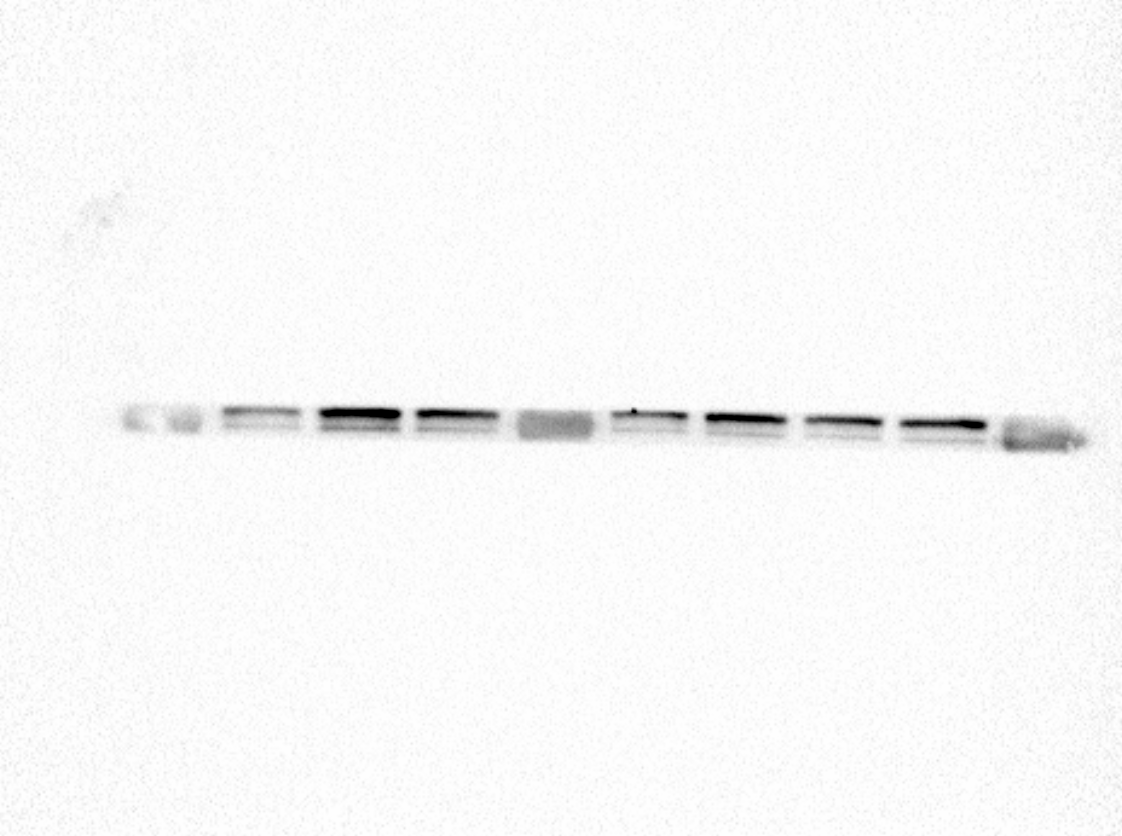

Supplement: Supplementary file 3 [file datasheet3.zip › NLRP3-original images/NLRP3-6-Administrator 2020-07-11 10 ╩▒ 03 ╖╓_Exposure_15.8sec.tif]

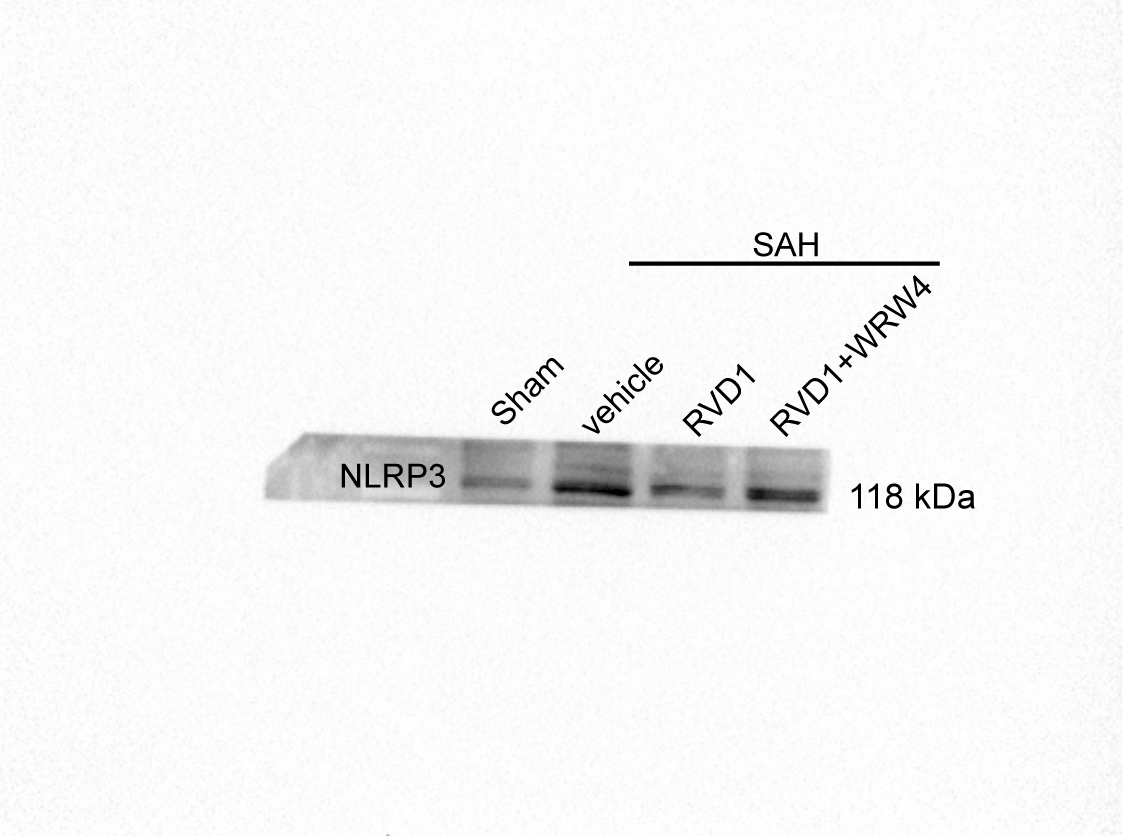

Supplement: Supplementary file 3 [file datasheet3.zip › NLRP3-original images/NLRP3▒Ω╫ó/NLRP3-1-Administrator 2020-06-13 09 ╩▒ 19 ╖╓_Exposure_31.1sec.tif]

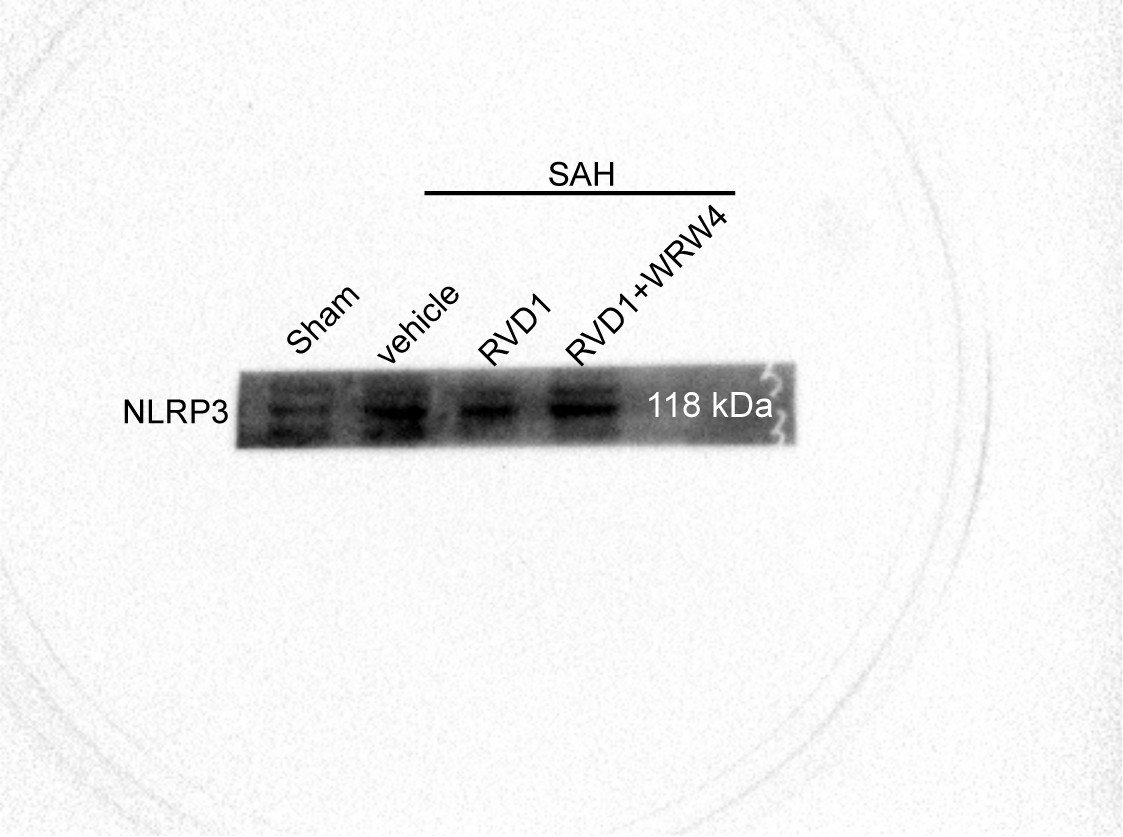

Supplement: Supplementary file 3 [file datasheet3.zip › NLRP3-original images/NLRP3▒Ω╫ó/NLRP3-2-Administrator 2020-06-17 08 ╩▒ 17 ╖╓_Exposure_67.7sec.tif]

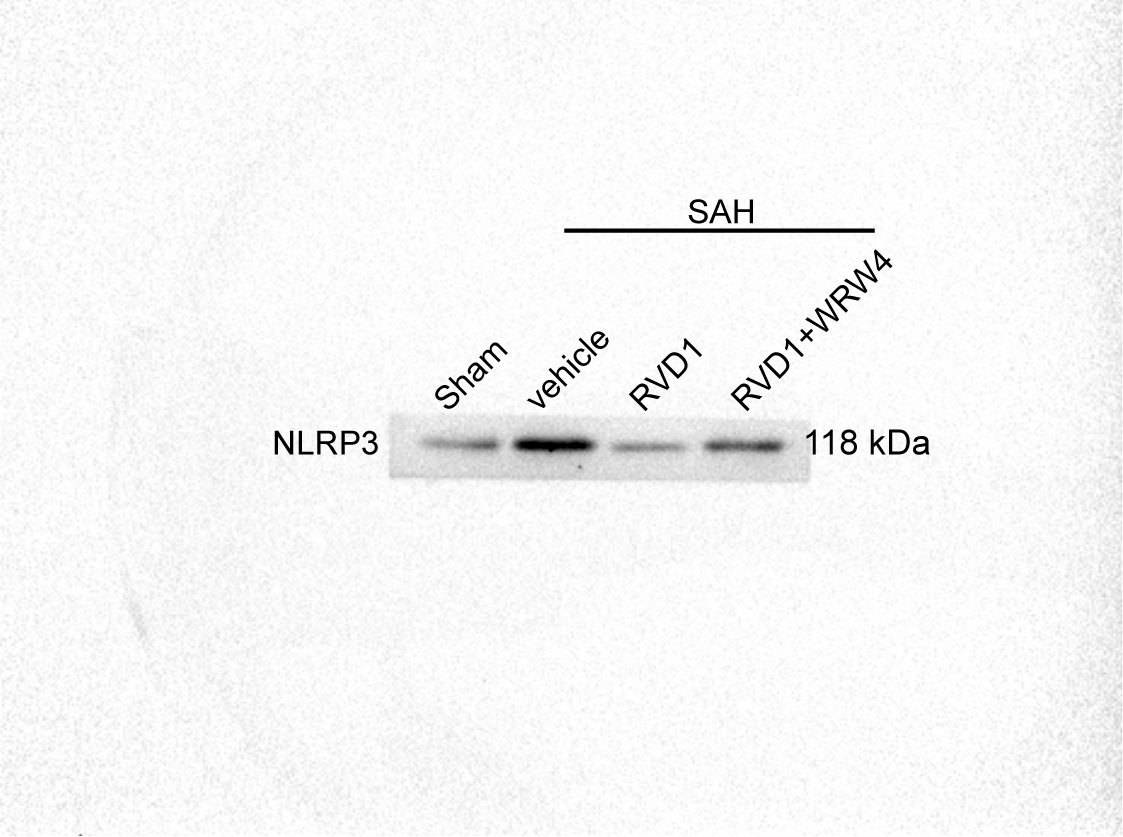

Supplement: Supplementary file 3 [file datasheet3.zip › NLRP3-original images/NLRP3▒Ω╫ó/NLRP3-3-Administrator 2020-06-17 23 ╩▒ 05 ╖╓_Exposure_133.9sec.tif]

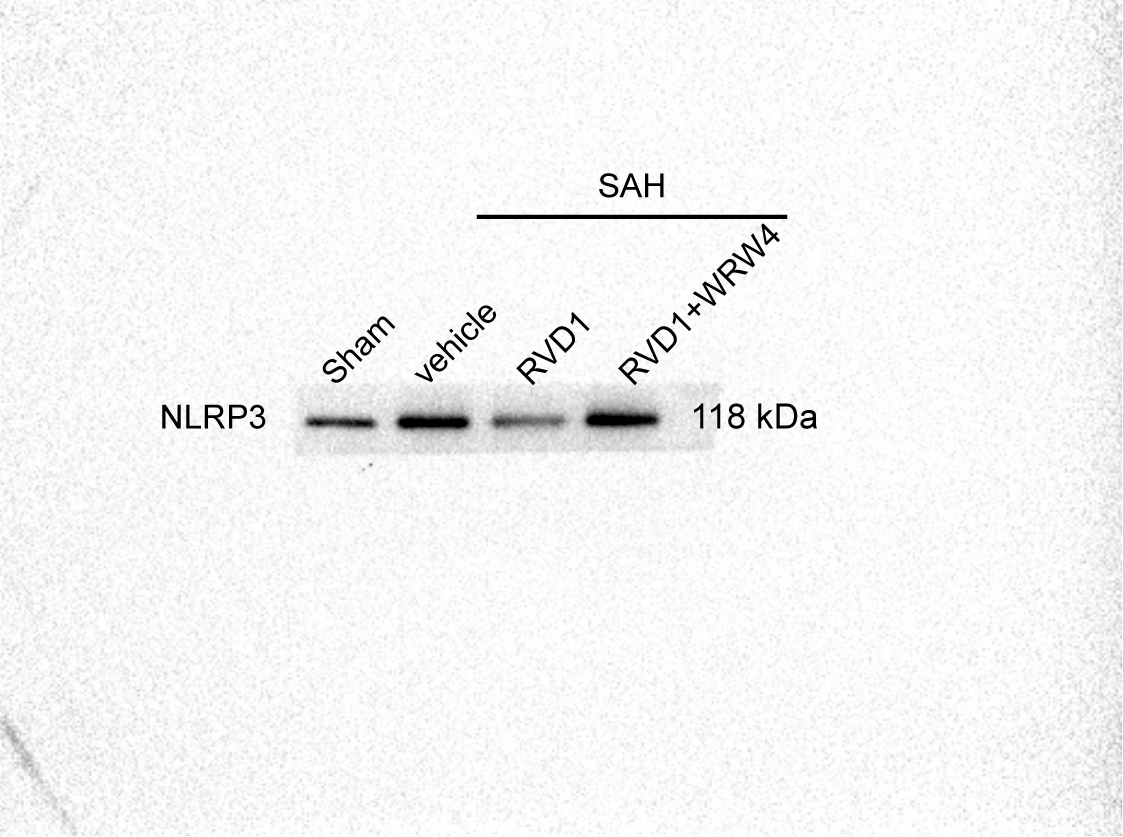

Supplement: Supplementary file 3 [file datasheet3.zip › NLRP3-original images/NLRP3▒Ω╫ó/NLRP3-4-Administrator 2020-06-18 23 ╩▒ 15 ╖╓_Exposure_59.3sec.tif]

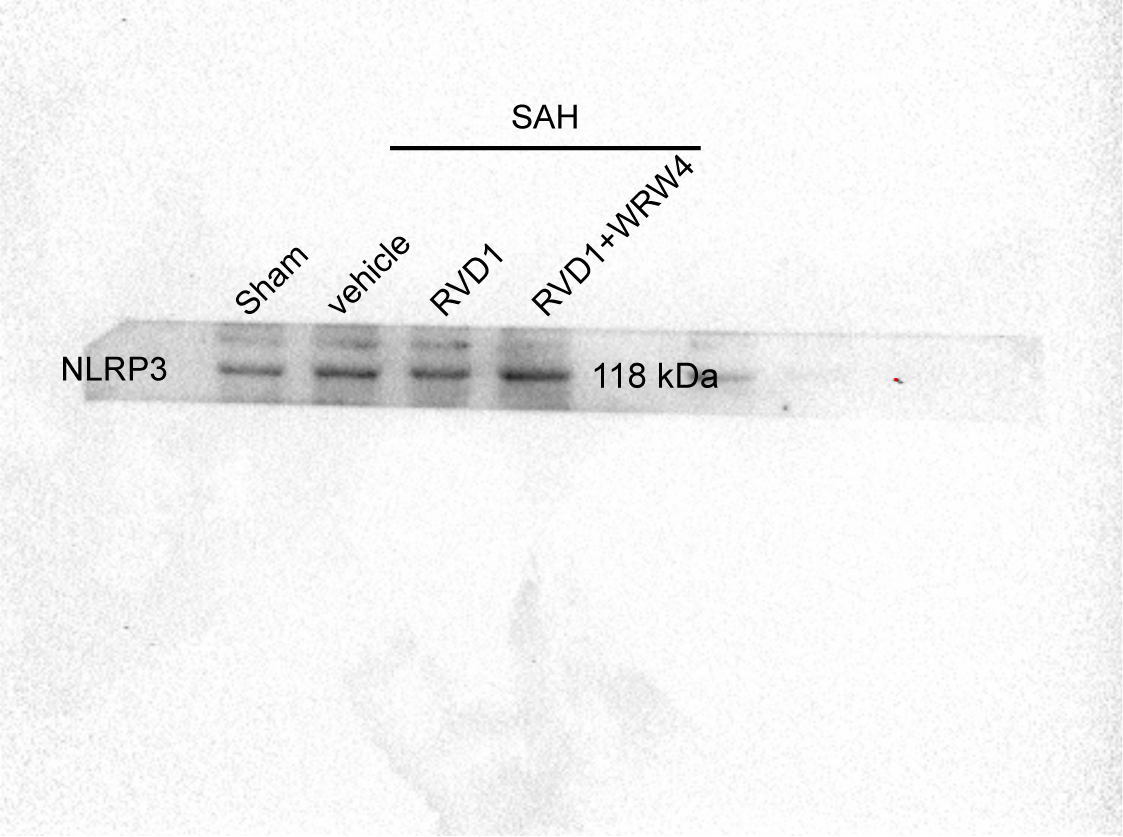

Supplement: Supplementary file 3 [file datasheet3.zip › NLRP3-original images/NLRP3▒Ω╫ó/NLRP3-5-Administrator 2020-06-19 00 ╩▒ 30 ╖╓_Exposure_222.6sec.tif]

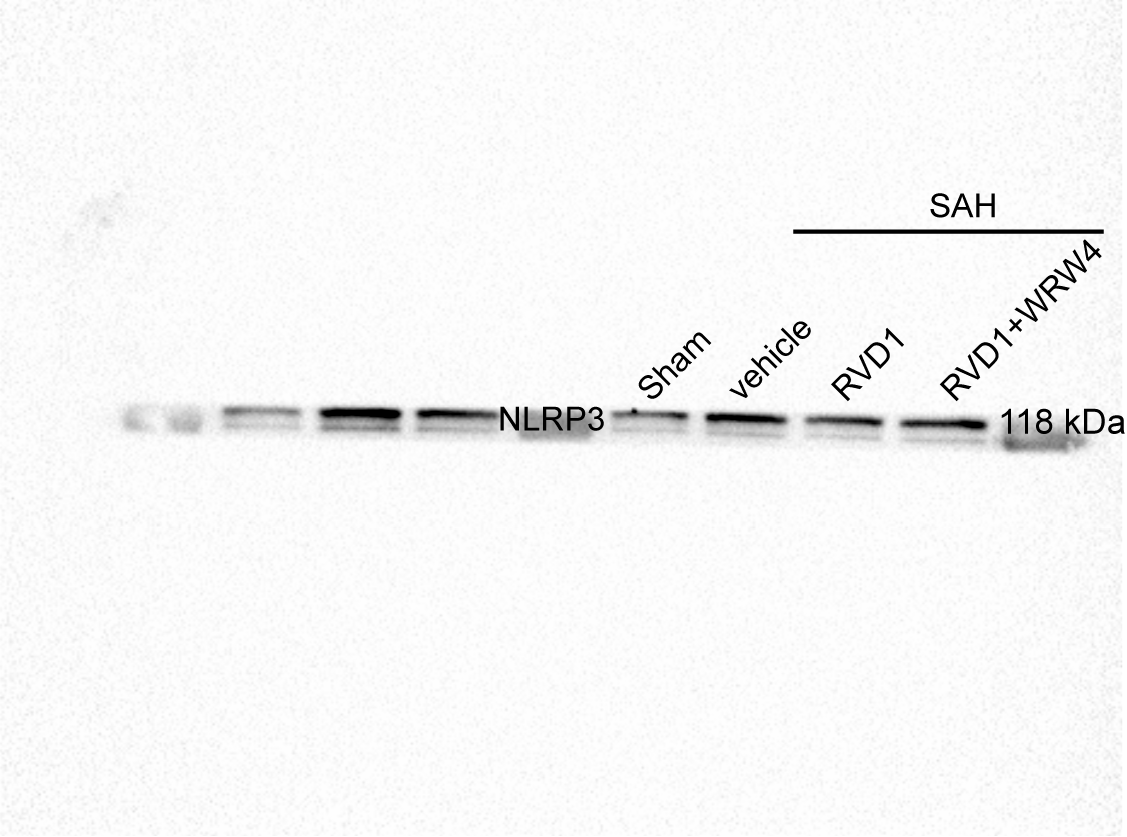

Supplement: Supplementary file 3 [file datasheet3.zip › NLRP3-original images/NLRP3▒Ω╫ó/NLRP3-6-Administrator 2020-07-11 10 ╩▒ 03 ╖╓_Exposure_15.8sec.tif]

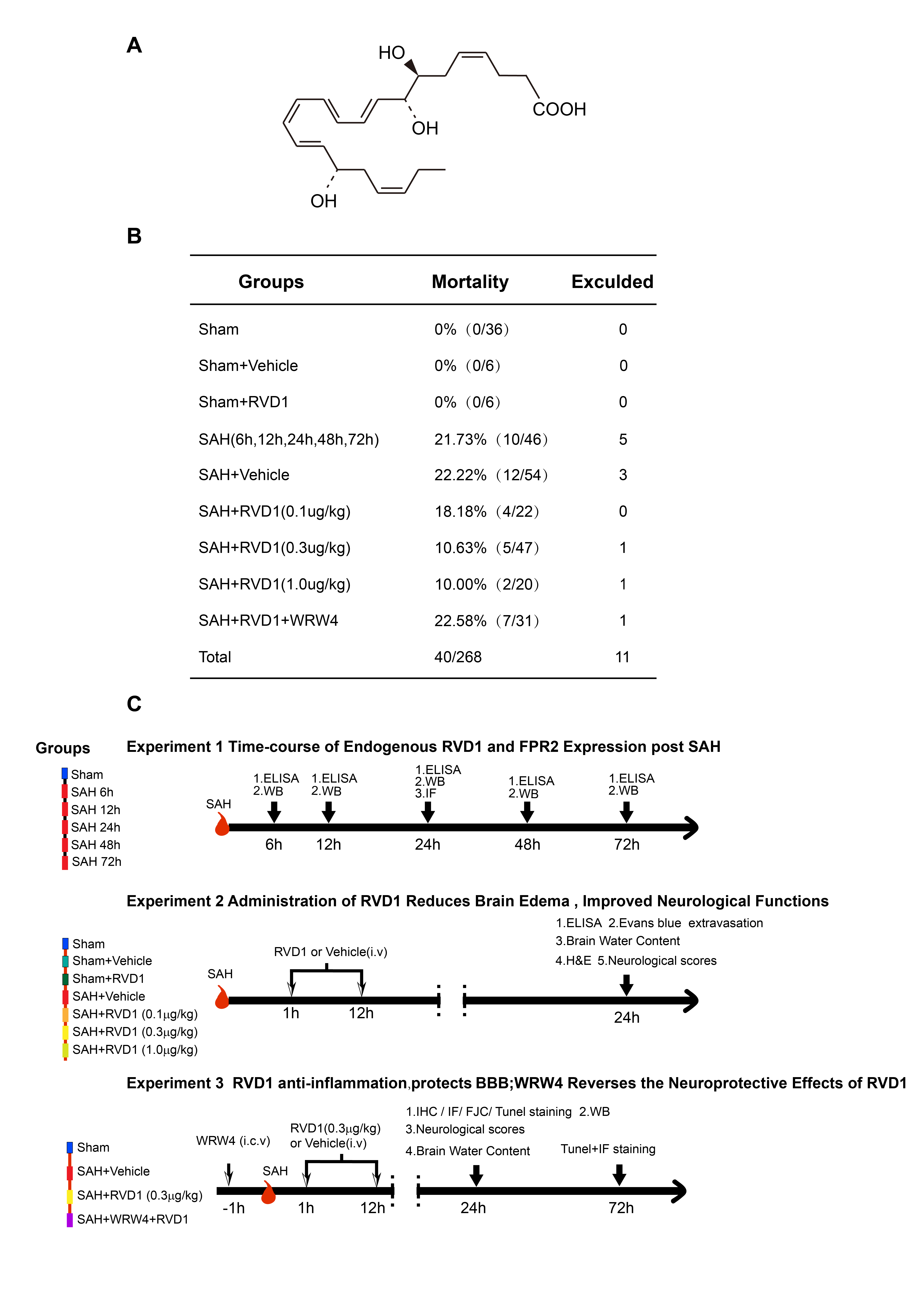

Supplement: Supplementary file 4 [file image1.jpg]

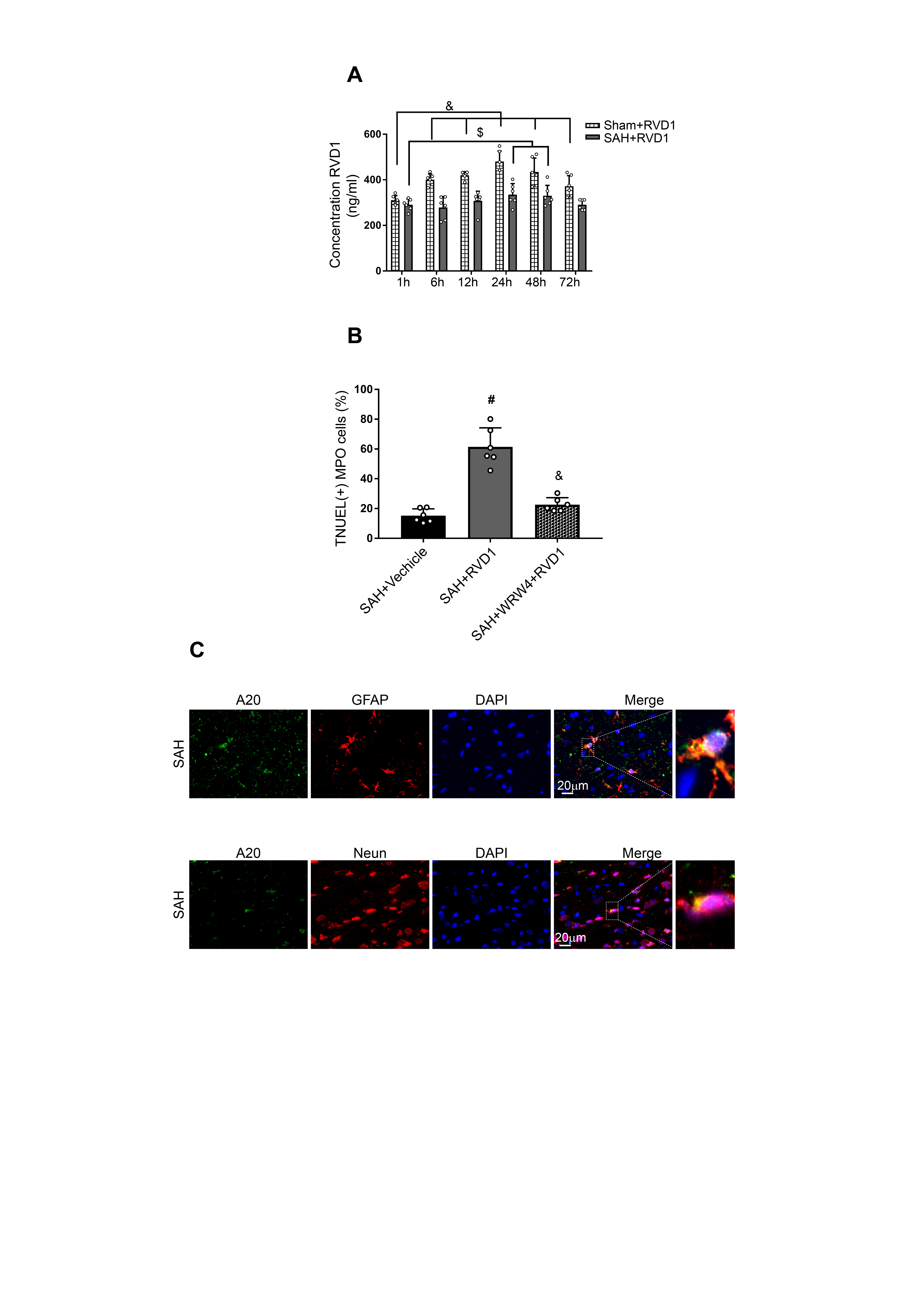

Supplement: Supplementary file 5 [file image2.jpeg]
